# Exploration of the System-Level Mechanisms of the Herbal Drug FDY003 for Pancreatic Cancer Treatment: A Network Pharmacological Investigation

**DOI:** 10.1155/2022/7160209

**Published:** 2022-05-10

**Authors:** Ho-Sung Lee, In-Hee Lee, Kyungrae Kang, Sang-In Park, Minho Jung, Seung Gu Yang, Tae-Wook Kwon, Dae-Yeon Lee

**Affiliations:** ^1^The Fore, 33 Saemunan-ro 5ga-gil, Jongno-gu, Seoul 03170, Republic of Korea; ^2^Forest Hospital, 33 Saemunan-ro 5ga-gil, Jongno-gu, Seoul 03170, Republic of Korea; ^3^Forestheal Hospital, 173 Ogeum-ro, Songpa-gu, Seoul 05641, Republic of Korea; ^4^Forest Hospital, 129 Ogeum-ro, Songpa-gu, Seoul 05549, Republic of Korea; ^5^Kyunghee Naro Hospital, 67, Dolma-ro, Bundang-gu, Seongnam 13586, Republic of Korea

## Abstract

Pancreatic cancer (PC) is the most lethal cancer with the lowest survival rate globally. Although the prescription of herbal drugs against PC is gaining increasing attention, their polypharmacological therapeutic mechanisms are yet to be fully understood. Based on network pharmacology, we explored the anti-PC properties and system-level mechanisms of the herbal drug FDY003. FDY003 decreased the viability of human PC cells and strengthened their chemosensitivity. Network pharmacological analysis of FDY003 indicated the presence of 16 active phytochemical components and 123 PC-related pharmacological targets. Functional enrichment analysis revealed that the PC-related targets of FDY003 participate in the regulation of cell growth and proliferation, cell cycle process, cell survival, and cell death. In addition, FDY003 was shown to target diverse key pathways associated with PC pathophysiology, namely, the PIK3-Akt, MAPK, FoxO, focal adhesion, TNF, p53, HIF-1, and Ras pathways. Our network pharmacological findings advance the mechanistic understanding of the anti-PC properties of FDY003 from a system perspective.

## 1. Introduction

Pancreatic cancer (PC) is the most lethal cancer type (0.47 million deaths per year worldwide) with the lowest survival rates [1]. At present, the standard anticancer drugs for PC treatment are mainly cytotoxic chemotherapeutics such as gemcitabine and FOLFIRINOX [2, 3]. However, these agents have limited effects on improving the survival rate of patients with PC, and their use is inevitably accompanied by toxic effects and drug resistance that have serious adverse effects on the physical and mental status of cancer patients [2–4]. Herbal drugs have been extensively studied and are increasingly recognized as effective anticancer agents that enhance the success rate of cancer treatment and inhibit the development of therapeutic resistance and unwanted side effects [5–8]. They were shown to play beneficial roles in improving survival and prognostic outcomes and health status, while reducing the mortality rate of patients with PC [5–8].

FDY003, which is made up of *Lonicera japonica* Thunberg (LjT), *Cordyceps militaris* (Cm), and *Artemisia capillaris* Thunberg (AcT), is an herbal drug that exerts anticancer activity [9–11]. This herbal drug has antiproliferative and apoptosis-promoting properties in a variety of cancer types by pharmacologically modulating cancer-associated genes and proteins that regulate and promote key protumorigenic cellular processes [9–11]. However, the anti-PC potential of FDY003 and the underlying mechanisms remain to be investigated.

Network pharmacology, an analysis methodology that aims to dissect the complicated multiple component-multiple target mechanisms of herbal drugs by integrating large-scale pharmacological information associated with them, is recognized as the most effective research technique for studying the therapeutic features of herbal drug [12–19]. Network pharmacology explores the therapeutic mechanisms of herbal drugs through the analysis of the topology, structures, and functions of various herbal drug-related networks that are composed of interactions between the phytochemical components and the target genes and proteins [12–19]. In this study, we investigated the anti-PC activities of FDY003 and its underlying pharmacological mechanisms from a systemic perspective based on network pharmacology approaches.

## 2. Materials and Methods

### 2.1. Cell Culture

The PANC-1 (a human PC cell line) cells were obtained from the Korean Cell Line Bank (Seoul, Korea) and maintained in Dulbecco's modified Eagle's medium (WELGENE Inc., Daegu, Korea) that contained 10% fetal bovine serum (WELGENE Inc., Daegu, Korea), and 1% penicillin-streptomycin (Thermo Fisher Scientific, Inc., Waltham, MA, USA) in a humidified 5% CO_2_ atmosphere at 37°C.

### 2.2. Herbal Drug Preparation

All dried raw herbal medicines (e.g., AcT [6.25 g], Cm [6.25 g], and LjT [4.16 g]) were obtained from Hanpure Pharmaceuticals (Pocheon, Korea). The herbal medicines were ground, mixed, and suspended in 70% ethanol (500 mL) and refluxed at 80°C for 3 h to obtain the herbal extracts. After filtering and purifying the herbal extracts consecutively with 80% and 90% ethanol, the samples were lyophilized at −80°C, stored at −20°C, and dissolved in distilled water before the experiments.

### 2.3. Assessment of the Effect of the Drug Treatment on the Cell Viability

The effect of drug treatment on cell viability was assessed using water-soluble tetrazolium salt (WST-1) experiments. After seeding 1.0 × 10^4^ cells in a 96-well plate, we incubated them with FDY003 in the presence or absence of gemcitabine (Sigma-Aldrich, St. Louis, MO, USA) for 72 h and subsequently added WST-1 solution (Daeil Lab Service Co., Ltd.; Seoul, Korea) for 2 h in a humidified 5% CO_2_ atmosphere at 37°C. The resulting cell viability was calculated following the measurement of the absorbance at 450 nm using an xMark microplate absorbance spectrophotometer (Bio-Rad, Hercules, CA, USA).

### 2.4. Screening of the Active Phytochemical Components

We investigated the list of the phytochemical components present in FDY003 and their pharmacokinetic information from the Traditional Chinese Medicine Systems Pharmacology [20], Bioinformatics Analysis Tool for Molecular Mechanism of Traditional Chinese Medicine [21], and Anticancer Herbs Database of Systems Pharmacology [22]. Among the chemical constituents of FDY003, we determined those that are potentially pharmacoactive using their drug-likenesses, Caco-2 permeabilities, and oral bioavailability parameters that are commonly used in network pharmacology studies to identify the active components [14, 20, 23]. Drug-likeness is a criterion to explore the possibility of a chemical component to function as a pharmacological agent determined by its molecular, physical, chemical, and structural aspects [20, 24]. A drug-likeness factor of ≥0.18 (the mean value of the drug-likeness score of all available drugs) is a common determinant for druggability [20, 24]. Caco-2 permeability is a criterion to investigate the permeable capacity of a chemical component in the intestinal system [20, 25–27]. Caco-2 permeability of ≥−0.4 is a general determinant for suitable intestinal permeability in drug design and the development processes [28, 29]. Oral bioavailability is a criterion to assess the fraction of a chemical component delivered to the systemic circulation after oral administration, which subsequently allows the chemical component access to the desired pharmacological site of action at different tissues and/or organs in the human body [20, 30]. Oral bioavailability of ≥30% is a determinant for reasonable distribution and absorption capability in the body [20, 30]. Overall, we determined the active phytochemical components to be those with drug-likeness ≥0.18, Caco-2 permeability ≥−0.4, and oral bioavailability ≥30% [14, 20, 23].

### 2.5. Identification of Molecular Targets of FDY003

We investigated the simplified molecular input line entry system (SMILES) information on the bioactive components of FDY003 from the PubChem database [28]. This information was then read into PharmMapper [29], SwissTargetPrediction [31], Similarity Ensemble Approach [32], and Search Tool for Interactions of Chemicals [33], to obtain the FDY003 targets for *Homo sapiens*. A list of the genes and proteins associated with the PC pathomechanisms was obtained from Online Mendelian Inheritance in Man [34], DrugBank [35], Pharmacogenomics Knowledgebase [36], Comparative Toxicogenomics Database [37], Human Genome Epidemiology Navigator [38], GeneCards [39], Therapeutic Target Database [40], and DisGeNET [41] using “pancreatic cancer” as the search term.

### 2.6. Generation of FDY003-Associated Networks

A network is composed of nodes, which represent herbal medicines, phytochemical components, target genes and proteins, pathways, etc., and links/edges, which refer to the molecular, functional, and pharmacological interactions between nodes [42]. A degree indicates the number of links (or edges) of node [42]. The herbal medicine-phytochemical component-target (H-C-T) network consists of the connected links between the herbal constituents of FDY003, their active phytochemical components, and the targeted PC-related genes and proteins. The H-C-T pathway (H-C-T-P) network is generated by adding the pathway information to the H-C-T network, which is performed by linking the targets of the H-C-T network to their associated pathways. The protein-protein interaction (PPI) network consists of connected links between the PC-associated targets using the molecular, genetic, and functional interaction information obtained from STRING [43]. The generation, depiction, and analysis of all the networks were conducted using Cytoscape [44].

### 2.7. Analysis of Survival Outcome of Patients with Pancreatic Cancer

The relationship between the survival outcome of PC patients and the expression levels of FDY003 targets were assessed using the Kaplan–Meier Plotter [45], a widely used comprehensive online database that integrates large-scale gene expression profiles and the survival information of patients with various cancer types obtained from the Gene Expression Omnibus (GEO) [46], European Genome-Phenome archive (EGA) [47], and The Cancer Genome Atlas (TCGA) [48]. The survival analysis was performed using the auto-selected best cutoff, and the results with *p* < 0.05 (log-rank test) were considered statistically significant.

### 2.8. Determination of Functional Enrichment of the FDY003 Targets

Functional enrichment of the FDY003 targets in terms of gene ontology (GO) and pathway was determined by uploading them into g:Profiler [49].

### 2.9. Investigation of Molecular Docking Activity

The structural data for the phytochemical components of FDY003 and their interacting targets were collected from the RCSB Protein Data Bank [50] and PubChem [28], respectively. The binding affinities of the interactions between the phytochemical components and the targets were determined using the scores for their molecular docking calculated using AutoDock Vina [51]. The phytochemical component-target pairs with molecular docking scores of ≤−5.0 were considered to have high binding affinities [52, 53].

## 3. Results

### 3.1. Exploration of the Inhibitory Activity of FDY003 on Pancreatic Cancer

To determine the anticancer properties of FDY003 for PC, we monitored the changes in the viability of PANC-1 cells treated with FDY003 in the presence or absence of gemcitabine, an anticancer drug clinically used for treating PC [54]. We found that FDY003 decreased the viability of PANC-1 cells and further enhanced the antiproliferative effect of gemcitabine (Supplementary Figure S1(a) and S1(b)), indicating the anti-PC potential of FDY003.

### 3.2. Identification of the Active Phytochemical Components of FDY003 and Their Interacting Therapeutic Targets

We considered the active phytochemical components of FDY003 as those satisfying the following parameters: drug-likeness ≥0.18, Caco-2 permeability ≥−0.4, and oral bioavailability ≥30%, as previously suggested (Supplementary Table S1) [9, 14, 20, 23]. In addition, some of them were included in the list of active components despite not fully meeting the corresponding requirements. Thus, 18 phytochemical components were found to be active in FDY003 (Supplementary Table S2). Afterward, we obtained 270 therapeutic targets based on the structural information of the active components of the herbal drug of which 123 were associated with PC pathophysiology (Supplementary Table S3).

### 3.3. Network-Based Investigation of FDY003 Mechanisms for Pancreatic Cancer Treatment

**Figure 1:**
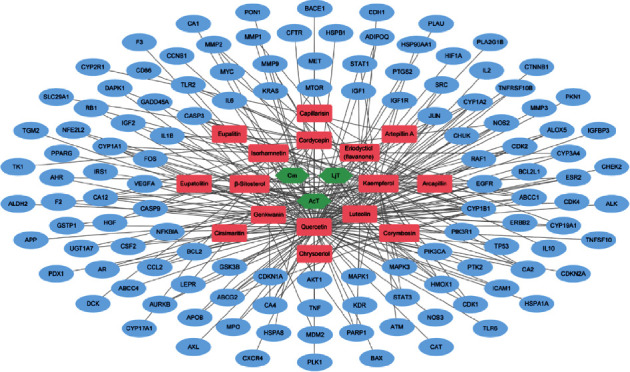
The herbal medicine-phytochemical component-target network for FDY003. Green nodes, herbal medicines; red nodes, active phytochemical components; blue nodes, pancreatic cancer-associated targets.

**Figure 2:**
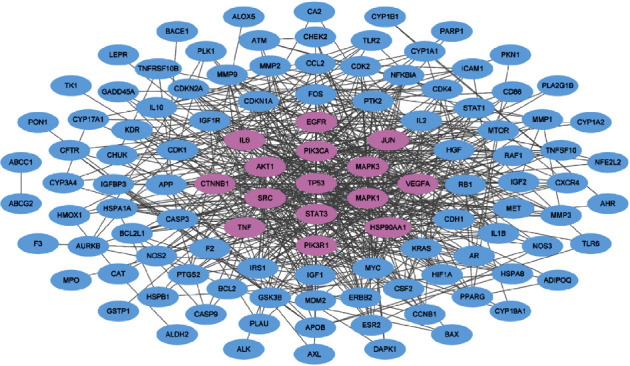
The protein-protein interaction network for the pancreatic cancer-associated targets of FDY003. Purple nodes, hub targets.

To conduct a network-based investigation of the anti-PC mechanisms of FDY003, we built an H-C-T network by merging and integrating large-scale FDY003-associated data. The network contains 142 nodes (three herbal medicines, 16 active components, and 123 PC-associated targets) and 261 links between them (Figure 1 and Supplementary Table S3). Quercetin, luteolin, and kaempferol were the components with the largest number of targets (Figure 2 and Supplementary Table S3), implying their crucial role in conferring the pharmacological effects on FDY003. Of note, 48.8% of the targets (60 out of 123 targets) were targeted by two or more phytochemical components (Figure 1), which suggests multiple component-multiple target polypharmacological activities of FDY003.

**Figure 3:**
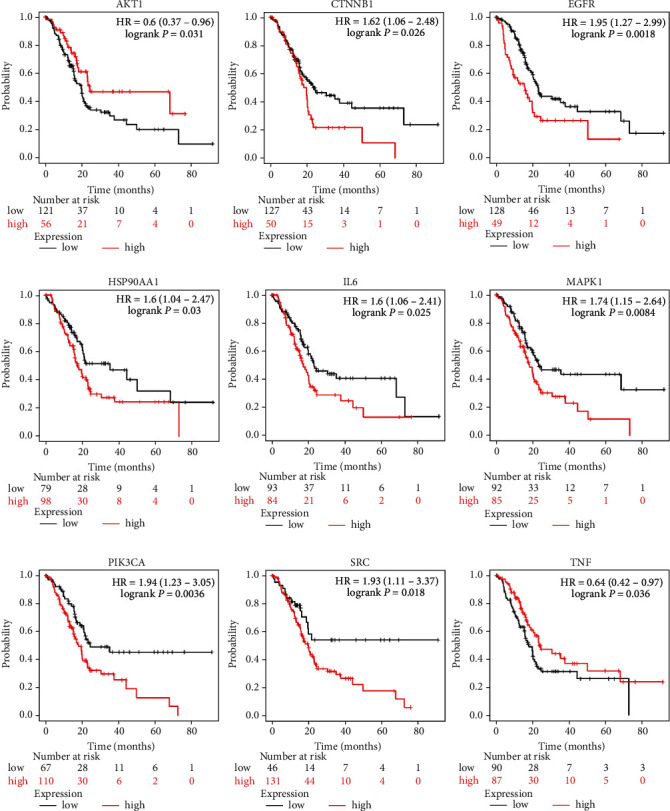
Survival analysis of the FDY003 targets. Kaplan–Meier curves analyzing the survival of patients with pancreatic cancer according to the expression levels of the indicated FDY003 targets.

Because the drugs exhibit their therapeutic activities by modulating the interactions with genes and proteins associated with disease mechanisms [55–59], we generated a PPI network using the PC-associated targets of FDY003 as nodes (Figure 2). By investigating the topological features of the PPI network, we identified high-degree hub nodes that are reported to exert functional significance and act as effective drug targets [60, 61]. According to previous findings, nodes were determined to be hubs if their degrees were twice or greater than the average node degree [62, 63]. Thus, the hubs in the network were AKT1, CTNNB1, EGFR, HSP90AA1, IL-6, JUN, MAPK1, MAPK3, PIK3CA, PIK3R1, SRC, STAT3, TNF, TP53, and VEGFA in the PPI network (Figure 2); this result suggests that these targets may play key roles in mediating the anti-PC effects of FDY003. We further found that the hub targets are potential predictors of the survival rates of patients with PC (Figure 3), which suggests their prognostic importance.

### 3.4. Functional Investigation of Antipancreatic Cancer Mechanisms of FDY003

**Figure 4:**
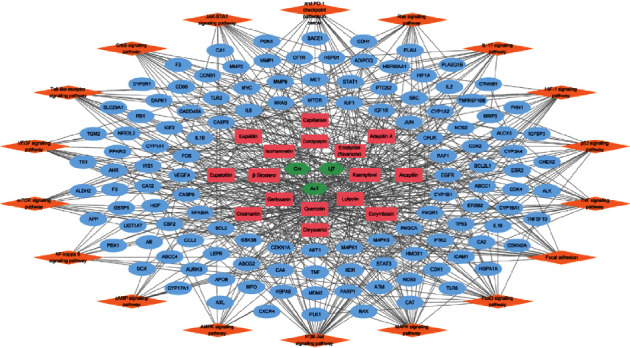
The herbal medicine-phytochemical component-target-pathway network for FDY003. Green nodes, herbal medicines; red nodes, active phytochemical components; blue nodes, pancreatic cancer-associated targets; orange nodes, pancreatic cancer-associated pathways.

To dissect the system-level mechanisms that underlie the therapeutic activities of FDY003 against PC from the perspective of molecules and pathways, we investigated the functional enrichment of PC-associated targets of FDY003. The analysis indicated that the targets may participate in the regulation and coordination of cell growth and proliferation, cell cycle process, survival, and apoptosis (Supplementary Figure S2). Moreover, the FDY003 targets acted as key components of various pathways related to the pathophysiology and signaling mechanisms of PC (Figure 4 and Supplementary Figure S2).

These functional analysis results show the system-level mechanisms underlying the anti-PC activity of FDY003 from the molecular- and pathway-level points of view.

### 3.5. Investigation of Binding Activities between the FDY003 Targets and Their Interacting Phytochemical Components Using a Molecular Docking Analysis

**Figure 5:**
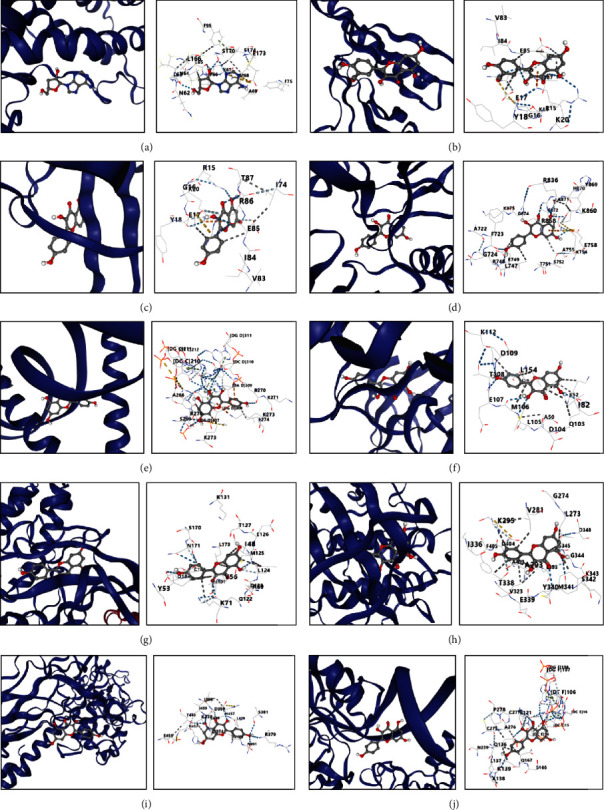
Molecular docking assessment of the pancreatic cancer-associated targets and the interacting active phytochemical components of FDY003. (a) Cordycepin-IL-6 (score = −6.1). (b) Isorhamnetin-AKT1 (score = −6.4). (c) Kaempferol-AKT1 (score = −6.9). (d) Kaempferol-EGFR (score = −8.1). (e) Kaempferol-JUN (score = −9.3). (f) Kaempferol-MAPK1 (score = −7.3). (g) Kaempferol-MAPK3 (score = −8.4). (h) Kaempferol-SRC (score = −8.6). (i) Kaempferol-STAT3 (score = −7.8). (j) Kaempferol-TP53 (score = −8.9).

To determine the binding affinities between the targets of FDY003 and their interacting phytochemical components, we conducted a molecular docking analysis. The analysis results indicated that the docking scores of phytochemical components and their hub targets were less than −5.0 (Figure 5 and Supplementary Figure S3), implying their strong binding potential.

## 4. Discussion

Although the therapeutic use of herbal drugs against PC has drawn growing attention [5–8], their complex polypharmacological properties have not been clearly understood. Here, we investigated the anti-PC activities of FDY003 and its underlying mechanisms in a systematic manner based on network pharmacology approaches. FDY003 decreased the viability of human PC cells and strengthened their pharmacological responses to chemotherapeutic agents. Network pharmacological analysis indicated that FDY003 possesses 16 active phytochemical components and 123 PC-related pharmacological targets. Functional enrichment analysis revealed that the PC-related targets of FDY003 were found to participate in the regulation of cell growth and proliferation, cell cycle process, survival, and cell death. In addition, FDY003 was shown to target diverse key pathways associated with PC pathophysiology, namely, the PIK3-Akt, MAPK, FoxO, focal adhesion, TNF, p53, HIF-1, and Ras pathways. Overall, the findings suggest that the anticancer effectiveness and underlying pharmacological mechanisms of FDY003 are potentially suitable for PC treatment.

The key target genes and proteins of FDY003 are reported to be strongly linked to PC pathomechanisms and potentially effective targets for PC therapeutics. The uncontrolled activation of the oncogenic kinase, Akt1 (encoded by *AKT1*), promotes pancreatic tumorigenesis and cancer progression; this kinase is a potential therapeutic target, and its expression levels and genetic polymorphisms are related to the survival, prognosis, and onset of cancer-associated disorders in patients with PC [64–69]. *β*-Catenin (encoded by *CTNNB1*) is upregulated in the tumor tissues of patients with PC and induces carcinogenesis, invasiveness, metastasis, angiogenesis, and therapeutic sensitivity of PC cells and tumors [70–74]. The oncogenic receptor tyrosine kinase epidermal growth factor receptor (EGFR; encoded by *EGFR*) is a predictor of sensitivity to anticancer agents and prognosis in patients with PC, and targeting it may suppress the angiogenesis, growth, metastasis, proliferation, and stemness of PC cells and tumors [75–78]. HSP90 (encoded by *HSP90AA1*) plays a role in the development of therapeutic resistance in PC, which can be overcome if targeted sufficiently [79, 80]. The pro-inflammatory cytokine interleukin (IL)-6 (encoded by *IL-6*) participates in the modulation of proliferation, migration, invasion, growth, oncogenesis, malignant progression, therapeutic resistance, tumor microenvironment, and remodeling of PC cells and tumors; the cytokine is further correlated with the survival, prognosis, tumor aggressiveness and metastasis, and occurrence of cancer-related complications of patients with PC [81–86]. The proto-oncogene c-Jun (encoded by *JUN*) is a potent contributor to the chemoresistance of PC, and its pharmacological modulation can enhance the sensitivity of anticancer therapeutics [87–89]. Targeting the extracellular signal-regulated kinase 1 (ERK1; encoded by *MAPK3*) and ERK2 (ERK2; encoded by *MAPK1*) may suppress diverse protumorigenic cellular phenotypes such as carcinogenesis, proliferation, angiogenesis, survival, metastasis, migration, invasion, and epithelial-to-mesenchymal transition (EMT) of PC cells and further alleviate resistance to anoikis and chemotherapeutics [90–95]. In addition, the expression levels of phosphorylated Akt and ERK may be associated with therapeutic and prognostic implications in patients with PC [96]. *PIK3CA* is involved in the initiation, migration, invasion, progression, and chemoresistance of PC cells [97–100]. *PI3KR1* expression is associated with lymphangiogenesis, lymphatic metastasis, and survival in PC [101, 102]. Src (encoded by *SRC*) is an oncogenic kinase that regulates EMT, migration, invasion, cell adhesion and spreading, metastasis, stem-like features, proliferation, growth, angiogenesis, and survival of PC cells, and its expression and activity are further correlated with the survival, progression, and therapy response rate of patients with PC [103–114]. Signal transducer and activator of transcription (STAT)-3 (encoded by *STAT3*) induces initiation, progression, proliferation, metastasis, angiogenesis, EMT, self-renewal stemness, migration, invasion, immune escape, and resistance to anoikis, chemotherapy, and radiotherapy of PC cells, and its activation is associated with poor prognosis of patients with PC [115–121]. Tumor necrosis factor*-α* (TNF-*α*; encoded by *TNF*) plays a crucial role in the pathological process of PC by regulating angiogenesis, metastasis, proliferation, pro-tumorigenic inflammation, chemoresistance, and immune evasion of PC cells, and increased expression levels are associated with enhanced cancer risk, tumor stage, lymph node metastases, cancer-associated symptoms, and poor prognosis of patients with PC [122–126]. The genetic and activity status of *TP53* is a key determinant of survival outcomes, recurrence, and disease progression of PC [127–129]. Vascular endothelial growth factor-A (VEGF-A; encoded by *VEGFA*) is closely involved in the angiogenesis of PC cells, and previous clinical studies have reported the relationship between its expression status with cancer grade and stage, tumor aggressiveness and metastasis, and prognostic and survival outcomes of patients with PC [130–134].

FDY003-targeted signalings are crucial pathways in PC pathomechanisms. The 5′ adenosine monophosphate-activated protein kinase (AMPK) and mammalian target of rapamycin (mTOR) pathways contribute to PC carcinogenesis and its malignant progression by enhancing survival, invasiveness, angiogenesis, proliferative growth, EMT, chemo- and radio-sensitivity, autophagy, stemness, and immune evasiveness of PC cells [135–140]. The cyclic adenosine monophosphate (cAMP) pathway regulates various protumorigenic processes such as migration, invasion, cell cycle progression, proliferation, stem-like ability, and metastasis of PC cells [141–144]. The oncogenic erythroblastic leukemia viral oncogene homolog (ErbB), focal adhesion, mitogen-activated protein kinase, phosphoinositide 3-kinase (PI3K)-Akt, and Ras signaling pathways are the key pathways responsible for the various mechanisms involved in the pathological processes of PC cells and tumors, and they have important roles as efficacious targets and biomarkers for survival and therapeutic response rate in patients with PC [145–151]. The forkhead box O (FoxO) pathway modulates stem cell-like and tumorigenic properties, metastatic potential, anchorage-independent growth capacity, and EMT of PC cells, and the loss of its expression is associated with carcinogenesis, large tumor mass, tumor invasion and metastasis, and shorter survival time of patients with PC [152–156]. The hypoxia-inducible factor-1 alpha (HIF-1*α*) pathway is a crucial regulator of cellular adaptation of PC cells to hypoxia, and its abnormal activity may induce tumorigenesis and development of PC by promoting uncontrolled survival and growth, metabolic reprogramming, desmoplasia, immune evasion, autophagy, EMT, invasion and metastasis, stem-like tumorigenicity, angiogenesis, and radioresistance and chemoresistance of PC cells [157, 158]. The IL-17 pathway accelerates tumorigenic and metastatic potential, and its activity serves as a predictor of PC prognosis and the efficacy of anticancer agents [159–162]. The Janus kinase (JAK)/STAT pathway is involved in carcinogenesis, development, growth, metastatic and angiogenic behaviors, immune surveillance, growth, stemness, anoikis resistance, EMT, treatment resistance, and invasion and migration of PC cells, while its activation is related to the reduced survival of patients with PC [115–121, 163, 164]. Dysregulation of the nuclear factor kappa B (NF-*κ*B) pathway is associated with reduced survival of patients with PC, and it has been considered as a promising target to suppress carcinogenicity, angiogenesis, malignant inflammation, metastasis, growth and proliferation, stem cell-like characteristics, and therapeutic resistance phenotypes of PC cells [165–167]. The genetic, epigenetic, transcriptional, translational, and post-translational loss of function of the tumor-suppressor p53 pathway may induce diverse cancerous cellular phenotypes of PC cells such as oncogenesis, metastasis, invasion, migration, EMT, proliferation, cell adaptation, and plasticity, and its functional restoration and activation not only inhibit the aforementioned pro-tumorigenic cellular processes but also induce antitumorigenic senescence and cell cycle and growth arrest of PC cells [119, 168–172]. Furthermore, the p53 pathway may have clinical significance because of its potential role as an indicator of progression, recurrence, and survival of patients with PC [127–129]. The programmed death-1 (PD-1)/programmed cell death ligand 1 (PD-L1) pathway is the primary site of cancer immunotherapy, and its activity and expression may predict prognosis, treatment sensitivity, immune response, invasion, metastasis to lymph nodes and distant sites, and appearance of adverse events and cancer symptoms of PC [173–177]. The TNF pathway contributes to not only the cancer-promoting inflammation of PC cells but also to their metastasis, immune surveillance, treatment resistant capacity, and angiogenic activities, and its expression and activation profiles are related to the risk of cancer induction, prognosis, metastasis, and cancer severity in patients with PC [122–126]. The toll-like receptor pathway participates in the coordination of cancerous cellular processes, including angiogenesis, stromal inflammation, tumorigenesis, proliferation, invasion, migration, angiogenesis, survival, and death of PC cells, and its signaling components are associated with the prognostic survival of patients with PC [178–183]. The VEGF pathway is the key target for antiangiogenic therapeutic strategies for PC treatment because of its potent protumorigenic angiogenic and metastatic properties [130–134].

The active phytochemical components of FDY003 have been previously shown to have potent anti-PC pharmacological roles. Chrysoeriol exerts pro-apoptotic effects on PC cells by targeting the survival-promoting protein B-cell lymphoma 2 (Bcl-2) [184]. Cordycepin inhibits the growth and survival of PC cells by modulating fibroblast growth factor receptor 2 (FGFR2), extracellular signal-regulated kinase (ERK), and mitochondrial signaling [185, 186]. Eriodyctiol (flavanone) inactivates the MAPK, FAK, and Akt pathways to induce cell cycle arrest and apoptosis of PC cells [187]. Isorhamnetin possesses antiproliferative, growth-arrest promoting, and antimigratory capabilities that are mediated by the regulation of the Ras/MAPK pathway in PC cells [188]. Kaempferol plays a crucial role in the regulation of the activities of Akt/mTOR, EGFR, ERK, Src, Bcl-2, caspase, PARP, TGM2, and ROS cascades, which leads to the suppression of survival, chemoresistance, and migration abilities of PC cells [189–192]. Luteolin induces apoptosis and chemosensitization but suppresses EMT, invasiveness, and angiogenesis of PC cells by targeting EGFR, Ras, GSK3-beta, IL-6, NF-*κ*B, STAT3, DPYD, VEGF, MMP, MMP, caspase, Bcl-2, PARP, and mitochondrial pathways [193–198]. Quercetin facilitates growth suppression, apoptosis, and chemosensitivity while inhibiting self-renewal ability, stemness, angiogenesis, metastasis, migration, invasion, proliferation, and EMT of PC cells, which are coordinated through the modulation of EGFR, RAGE/PI3K/Akt/mTOR, NF-*κ*B, ALDH1, EGFR, IL-6, STAT3, SHH, TGF-beta1, Smad2/3, Snail, and p-glycoprotein cascades [198–204]. *β*-Sitosterol targets NF-*κ*B, Bcl-2, and Akt/GSK3-beta pathways, and this effect downregulates EMT, growth, proliferation, cell cycle progression, and survival of PC cells [205].

## 5. Conclusion

In summary, our network pharmacology study provides a comprehensive understanding of the systematic mechanisms underlying the anti-PC effects of FDY003. FDY003 decreased the viability of human PC cells and strengthened their pharmacological responses to chemotherapeutic agents. Network pharmacological analysis of FDY003 revealed its active phytochemical components and their targeted genes, proteins, and PC-associated pathways, as well as the polypharmacological molecular mechanisms of the herbal drug. Further research is needed to determine the therapeutic role of FDY003 in key protumorigenic cellular processes such as cancer cell metastasis, angiogenesis, and self-renewal stemness potential to enlarge the application of herbal drugs as cancer therapeutics.

## Data Availability

The data used to support the findings of this study are included within the article.

## Supplementary Materials

Supplementary Materials Supplementary Figure S1: effects of FDY003 on the viability of human pancreatic cancer cells. Supplementary Figure S2: functional enrichment analysis for the pancreatic cancer-associated targets of FDY003. Supplementary Figure S3: molecular docking assessment of the pancreatic cancer-associated targets and the interacting active phytochemical components of FDY003. Supplementary Table S1: list of phytochemical components of FDY003. Supplementary Table S2: list of active phytochemical components of FDY003. Supplementary Table S3: list of targets of active phytochemical components of FDY003.
