# Supplementary material for: Exploration of the System-Level Mechanisms of the Herbal Drug FDY003 for Pancreatic Cancer Treatment: A Network Pharmacological Investigation
Source: Evid Based Complement Alternat Med. 2022 May 10;2022:7160209. doi: 10.1155/2022/7160209 (PMC9113891; doi:10.1155/2022/7160209)
Supplement: Supplementary Materials — Supplementary Figure S1: effects of FDY003 on the viability of human pancreatic cancer cells. Supplementary Figure S2: functional enrichment analysis for the pancreatic cancer-associated targets of FDY003. Supplementary Figure S3: molecular docking assessment of the pancreatic cancer-associated targets and the interacting active phytochemical components of FDY003. Supplementary Table S1: list of phytochemical components of FDY003. Supplementary Table S2: list of active phytochemical components of FDY003. Supplementary Table S3: list of targets of active phytochemical components of FDY003. [file 7160209.f1.docx]

**Supplementary Materials**

**Exploration of the system-level mechanisms of the herbal drug FDY003
for pancreatic cancer treatment: A network pharmacological investigation**

Ho-Sung Lee^1,2,*^, In-Hee Lee^1^, Kyungrae Kang^2^, Sang-In Park^3^, Minho Jung^4^, Seung Gu Yang^5^, Tae-Wook Kwon^2^, and Dae-Yeon Lee^1,2,*^

^1^ The Fore, 33 Saemunan-ro 5ga-gil, Jongno-gu, Seoul 03170, Republic of Korea.
^2^ Forest Hospital, 33 Saemunan-ro 5ga-gil, Jongno-gu, Seoul 03170, Republic of Korea.
^3^ Forestheal Hospital, 173 Ogeum-ro, Songpa-gu, Seoul 05641, Republic of Korea.
^4^ Forest Hospital, 129 Ogeum-ro, Songpa-gu, Seoul 05549, Republic of Korea.
^5^ Kyunghee Naro Hospital, 67, Dolma-ro, Bundang-gu, Seongnam 13586, Republic of Korea.

^*^Correspondence should be addressed to Ho-Sung Lee (forehslee@gmail.com)
and Dae-Yeon Lee (foresthrnd@gmail.com)

## Supplementary Figures


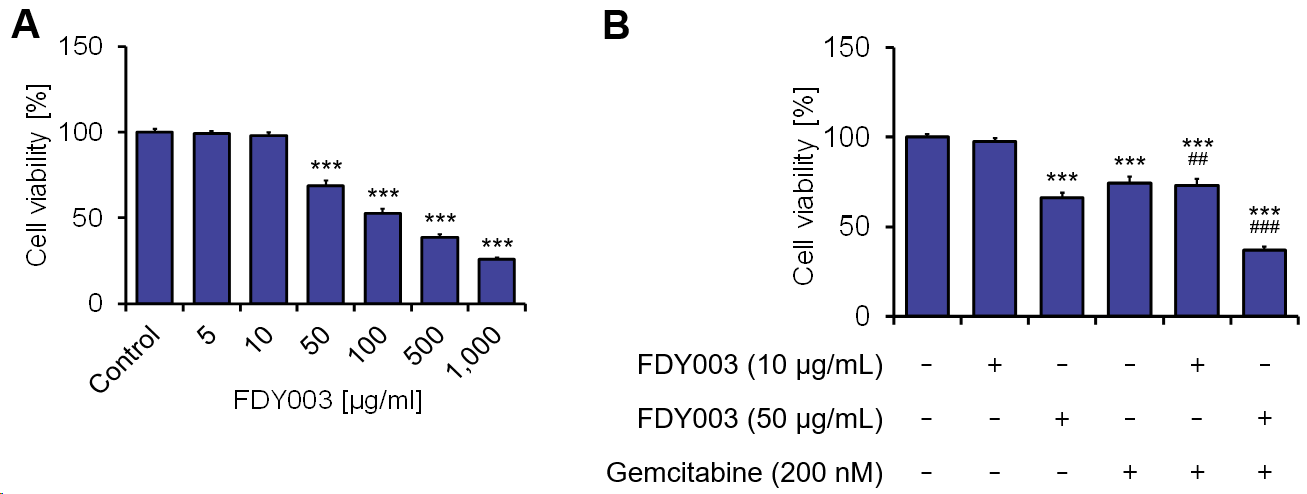


**Supplementary Figure S1. Effects of FDY003 on the viability of human pancreatic cancer cells. (A)** PANC-1 human pancreatic cancer cells were treated with indicated doses of FDY003 and their viability was measured. **(B)** PANC-1 human pancreatic cancer cells were treated with indicated doses of FDY003 and/or gemcitabine, a cytotoxic anticancer agent, for 72 hours and their viability was measured. Data represent the mean ± S.E.M. of five replicates. ***, p < 0.001; two-tailed Student’s t-test vs the untreated control cells. ##, p < 0.01; ###, p < 0.001; two-tailed Student’s t-test vs the gemcitabine-treated cells.


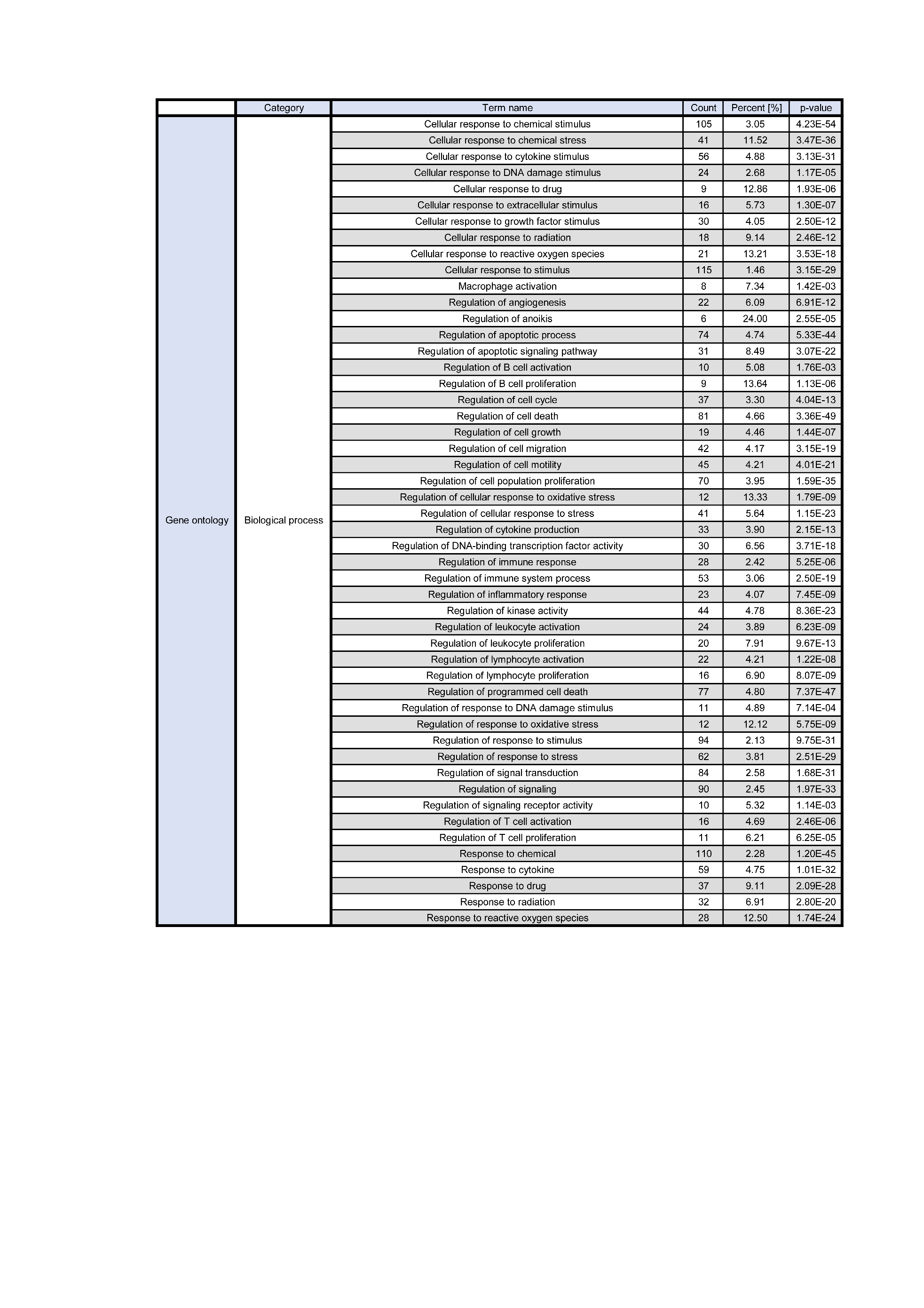


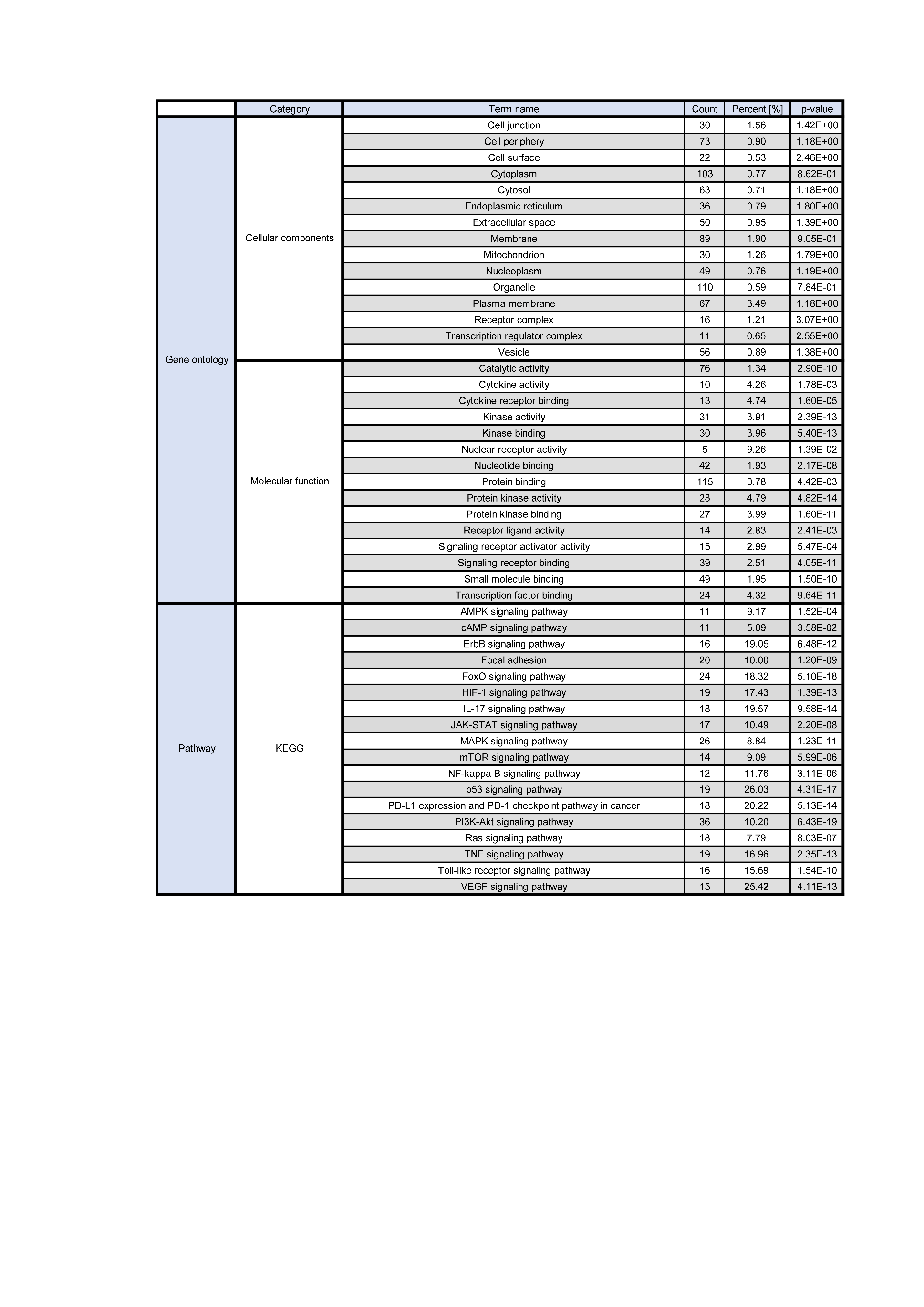


**Supplementary Figure S2. Functional enrichment analysis for the pancreatic cancer-associated targets of FDY003.** Tables showing the result of gene ontology and pathway enrichment analysis for the pancreatic cancer-associated targets of FDY003.


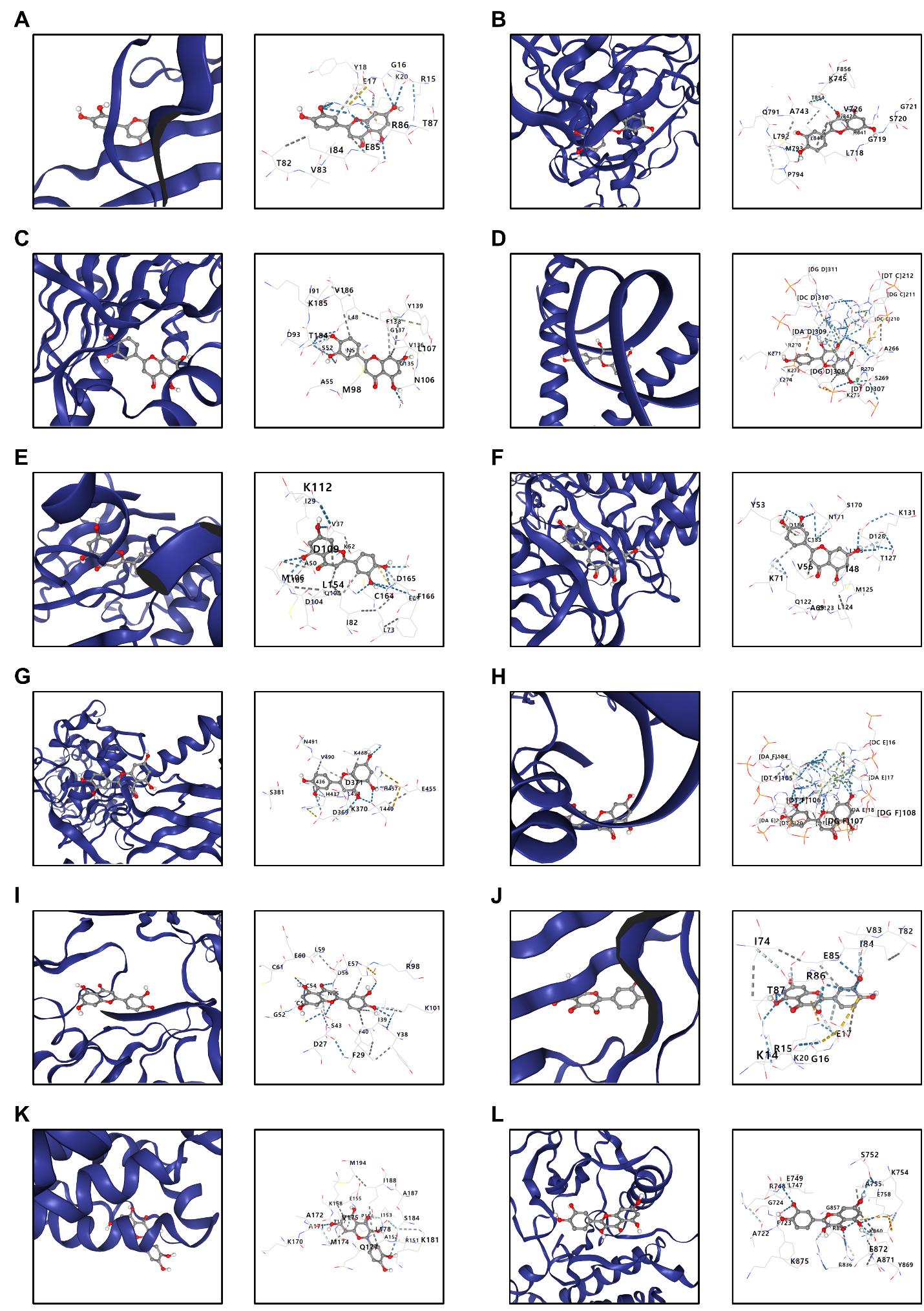


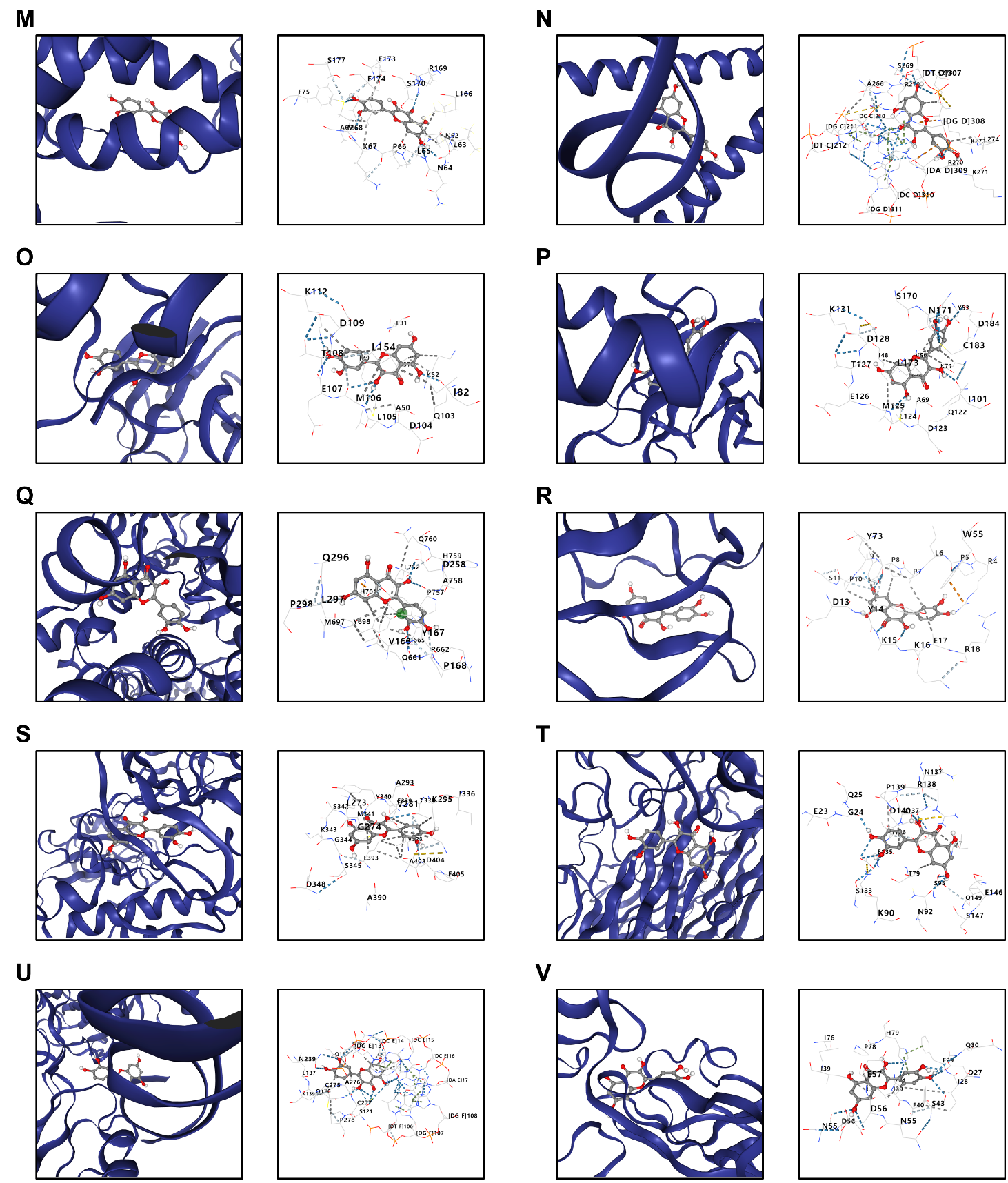


**Supplementary Figure S3.** **Molecular docking assessment of the pancreatic cancer-associated targets and the interacting active phytochemical components of FDY003. (A)** Luteolin-AKT1 (score = -6.6). **(B)** Luteolin-EGFR (score = -8.4). **(C)** Luteolin-HSP90AA1 (score = -7.7). **(D)** Luteolin-JUN (score = -9.0). **(E)** Luteolin -MAPK1 (score = -7.9). **(F)** Luteolin-MAPK3 (score = -7.4). **(G)** Luteolin-STAT3 (score = -8.0). **(H)** Luteolin-TP53 (score = -9.3). **(I)** Luteolin-VEGFA (score = -7.8). **(J)** Quercetin-AKT1 (score = -6.6). **(K)** Quercetin-CTNNB1 (score = -7.2). **(L)** Quercetin-EGFR (score = -8.2). **(M)** Quercetin-IL6 (score = -7.0). **(N)** Quercetin-JUN (score = -9.5). **(O)** Quercetin-MAPK1 (score = -7.3). **(P)** Quercetin-MAPK3 (score = -8.6). **(Q)** Quercetin-PIK3CA (score = -7.8). **(R)** Quercetin-PIK3R1 (score = -6.4). **(S)** Quercetin-SRC (score = -8.7). **(T)** Quercetin-TNF (score = -8.9). **(U)** Quercetin-TP53 (score = -9.1). **(V)** Quercetin-VEGFA (score = -8.3).

## Supplementary Tables

**Supplementary Table S1. List of phytochemical components of FDY003.**

| Herbal medicines | Phytochemical components | OB | Caco-2 | DL | Hdon | Hacc | MW | AlogP | RBN |
| --- | --- | --- | --- | --- | --- | --- | --- | --- | --- |
| LjT | (-)-(3R,8S,9R,9aS,10aS)-9-ethenyl-8-(beta-D-glucopyranosyloxy)-2,3,9,9a,10,10a-hexahydro-5-oxo-5H,8H-pyrano[4,3-d]oxazolo[3,2-a]pyridine-3-carboxylic acid | 3.22 | -1.72 | 0.80 | 5 | 12 | 443.45 | -2.71 | 5 |
| LjT | (-)-(3R,8S,9R,9aS,10aS)-9-ethenyl-8-(beta-D-glucopyranosyloxy)-2,3,9,9a,10,10a-hexahydro-5-oxo-5H,8H-pyrano[4,3-d]oxazolo[3,2-a]pyridine-3-carboxylic acid_qt | 87.47 | -0.55 | 0.23 | 2 | 7 | 281.29 | -0.96 | 2 |
| LjT | (-)-Caryophyllene oxide | 32.67 | 1.58 | 0.13 | 0 | 1 | 220.39 | 3.52 | 0 |
| LjT | ()-Menthol | 59.33 | 1.27 | 0.03 | 1 | 1 | 156.30 | 2.78 | 1 |
| LjT | (-)-α-Pinene | 46.25 | 1.85 | 0.05 | 0 | 0 | 136.26 | 2.87 | 0 |
| LjT | ()-α-Terpineol | 46.30 | 1.28 | 0.03 | 1 | 1 | 154.28 | 2.42 | 1 |
| LjT | (+)-Ledol | 16.96 | 1.43 | 0.12 | 1 | 1 | 222.41 | 3.20 | 0 |
| LjT | (1alpha,3R,4alpha,5R)-3,4,5-Tris[[(2E)-3-(3,4-dihydroxyphenyl)-1-oxo-2-propen-1-yl]oxy]-1-hydroxycyclohexanecarboxylic acid | 3.01 | -1.46 | 0.45 | 7 | 15 | 677.63 | 2.86 | 13 |
| LjT | (1R,2R,4R)-Dihydrocarveol | 51.17 | 1.32 | 0.03 | 1 | 1 | 154.28 | 2.58 | 1 |
| LjT | (1R,4S,4aR)-1-isopropyl-4-methyl-7-methylene-2,3,4,4a,5,6-hexahydro-1H-naphthalene | 19.50 | 1.84 | 0.08 | 0 | 0 | 204.39 | 4.80 | 1 |
| LjT | (1S,2S)-2-isopropenyl-4-isopropylidene-1-methyl-1-vinylcyclohexane | 34.47 | 1.87 | 0.06 | 0 | 0 | 204.39 | 4.93 | 2 |
| LjT | (1S,4E,8E,10R)-4,8,11,11-tetramethylbicyclo[8.1.0]undeca-4,8-diene | 21.69 | 1.86 | 0.08 | 0 | 0 | 204.39 | 4.70 | 0 |
| LjT | (2S)-2-methylbutan-1-ol | 81.23 | 1.06 | 0.00 | 1 | 1 | 88.17 | 1.29 | 2 |
| LjT | (3R,4aS,5R,6R)-6-hydroxy-3-methoxy-5-vinyl-4,4a,5,6-tetrahydro-3H-pyrano[5,4-c]pyran-1-one | 60.89 | 0.09 | 0.10 | 1 | 5 | 226.25 | -0.04 | 2 |
| LjT | (5Z,9Z)-6,10,14-trimethylpentadeca-5,9,13-trien-2-one | 37.84 | 1.58 | 0.10 | 0 | 1 | 262.48 | 5.45 | 9 |
| LjT | (E,E,E)-3,7-11,16-tetramethyl hexadeca-2,6,10,14-tetraen-1-ol | 42.48 | 1.42 | 0.14 | 1 | 1 | 290.54 | 6.60 | 11 |
| LjT | (E,Z)-farnesol | 36.73 | 1.32 | 0.06 | 1 | 1 | 222.41 | 4.76 | 7 |
| LjT | (S)-phenethyl 2-bromopropanoate | 9.10 | 1.27 | 0.05 | 0 | 2 | 257.14 | 3.19 | 5 |
| LjT | (Z,E)-farnesol | 41.14 | 1.34 | 0.06 | 1 | 1 | 222.41 | 4.76 | 6 |
| LjT | [(1S)-endo]-(-)-Borneol | 83.54 | 1.22 | 0.05 | 1 | 1 | 154.28 | 1.98 | 0 |
| LjT | 1,6-Dicyclohexylhexane | 15.40 | 1.81 | 0.11 | 0 | 0 | 250.52 | 7.43 | 7 |
| LjT | 1H,3H-Pyrano(3,4-c)pyran-1-one, 5-ethenyl-6-(beta-D-glucopyranosyloxy)-4,4a,5,6-tetrahydro-, (4aS-(4aalpha,5beta,6alpha))- | 4.96 | -1.08 | 0.38 | 4 | 9 | 358.38 | -1.59 | 4 |
| LjT | 2-(2,4-dimethoxyphenyl)-3-hydroxy-7-methoxy-chromone | 12.94 | 0.72 | 0.33 | 1 | 6 | 328.34 | 2.52 | 4 |
| LjT | 2-(3,4-dimethoxyphenyl)-5-hydroxy-7-methoxy-chromone | 29.24 | 0.90 | 0.34 | 1 | 6 | 328.34 | 2.82 | 3 |
| LjT | 2,3-DIMETHYLPYRAZINE | 30.82 | 1.07 | 0.02 | 0 | 2 | 108.16 | 0.09 | 0 |
| LjT | 2-[(1R,3S,4S)-3-isopropenyl-4-methyl-4-vinylcyclohexyl]propan-2-ol | 19.03 | 1.37 | 0.07 | 1 | 1 | 222.41 | 3.70 | 3 |
| LjT | 2-[(2S,5R)-5-ethenyl-5-methyloxolan-2-yl]propan-2-ol | 68.08 | 0.99 | 0.04 | 1 | 2 | 170.28 | 1.43 | 2 |
| LjT | 2-bromododecane | 17.60 | 1.82 | 0.03 | 0 | 0 | 249.27 | 5.89 | 9 |
| LjT | 2H-Pyran-5-carboxylic acid, 4-(2,2-dimethoxyethyl)-3-ethenyl-2-(beta-D-glucopyranosyloxy)-3,4-dihydro-, methyl ester, (2S,3R,4S)- | 22.59 | -1.25 | 0.45 | 4 | 11 | 434.49 | -1.73 | 10 |
| LjT | 2-isopropenyl-5-methylhex-4-enal | 27.69 | 1.33 | 0.02 | 0 | 1 | 152.26 | 2.83 | 4 |
| LjT | 2-methylpentadecane | 4.35 | 1.82 | 0.06 | 0 | 0 | 226.50 | 7.47 | 12 |
| LjT | 3,4-Dicaffeoylquinic acid | 1.71 | -1.40 | 0.69 | 6 | 12 | 515.48 | 0.88 | 9 |
| LjT | 3,4-Dimethyl-2-hexanone | 35.01 | 1.38 | 0.01 | 0 | 1 | 128.24 | 2.05 | 3 |
| LjT | 3,4-di-O-caffeoylquinic acid methyl ester | 1.71 | -1.02 | 0.69 | 6 | 12 | 530.52 | 1.81 | 10 |
| LjT | 3,5-di-O-caffeoylquinic acid methyl ester | 1.73 | -0.96 | 0.68 | 6 | 12 | 530.52 | 1.81 | 10 |
| LjT | 3-Hexenol | 62.74 | 1.05 | 0.01 | 1 | 1 | 100.18 | 1.44 | 3 |
| LjT | 3-Methyl-2-pent-2-enyl-cyclopent-2-enone | 25.28 | 1.52 | 0.03 | 0 | 1 | 164.27 | 2.97 | 3 |
| LjT | 3-O-Methylquercetin | 10.10 | 0.20 | 0.30 | 4 | 7 | 316.28 | 1.57 | 2 |
| LjT | 4,5-Dicaffeoylquinic acid | 1.73 | -1.37 | 0.69 | 6 | 12 | 515.48 | 0.88 | 9 |
| LjT | 4,5-di-O-caffeoylquinic acid methyl ester | 1.73 | -0.99 | 0.69 | 6 | 12 | 530.52 | 1.81 | 10 |
| LjT | 4,5'-Retro-.beta.,.beta.-Carotene-3,3'-dione, 4',5'-didehydro- | 31.22 | 1.17 | 0.55 | 0 | 2 | 562.90 | 9.27 | 9 |
| LjT | 4-caffeoylquinic acid | 10.48 | -1.52 | 0.33 | 5 | 9 | 353.33 | -1.09 | 5 |
| LjT | 4-stearylmorpholine | 14.80 | 1.48 | 0.27 | 0 | 2 | 339.68 | 7.72 | 17 |
| LjT | 5-O-Caffeoyl quinic acid butyl ester | 8.77 | -0.65 | 0.41 | 5 | 9 | 410.46 | 1.16 | 9 |
| LjT | 7-epi-Loganin | 4.78 | -1.65 | 0.44 | 5 | 10 | 390.43 | -2.08 | 5 |
| LjT | 7-epi-Loganin_qt | 85.12 | -0.28 | 0.10 | 2 | 5 | 228.27 | -0.33 | 2 |
| LjT | 7-epi-Vogeloside | 46.13 | -1.30 | 0.58 | 4 | 11 | 432.47 | -0.19 | 6 |
| LjT | 7-epi-Vogeloside_qt | 33.26 | 0.04 | 0.16 | 1 | 6 | 270.31 | 0.99 | 3 |
| LjT | 8-epiloganin | 11.68 | -1.74 | 0.44 | 5 | 10 | 390.43 | -2.08 | 5 |
| LjT | 8-epiloganin_qt | 26.42 | -0.64 | 0.10 | 2 | 5 | 228.27 | -0.33 | 2 |
| LjT | 9-epi-(E)-caryophyllene | 30.28 | 1.83 | 0.09 | 0 | 0 | 204.39 | 4.75 | 0 |
| LjT | Akebiasaponin D | 1.67 | -3.02 | 0.07 | 11 | 18 | 929.23 | 0.44 | 10 |
| LjT | Akebiasaponin D_qt | 16.44 | 0.19 | 0.74 | 3 | 4 | 472.78 | 5.33 | 2 |
| LjT | Alloaromadedrene | 53.46 | 1.83 | 0.10 | 0 | 0 | 204.39 | 4.22 | 0 |
| LjT | Amylol | 76.16 | 1.02 | 0.00 | 1 | 1 | 88.17 | 1.43 | 3 |
| LjT | Apigenin | 23.06 | 0.43 | 0.21 | 3 | 5 | 270.25 | 2.33 | 1 |
| LjT | Astragalin | 14.03 | -1.34 | 0.74 | 7 | 11 | 448.41 | -0.32 | 4 |
| LjT | Atractylodin | 44.49 | 2.00 | 0.05 | 0 | 1 | 182.23 | 3.83 | 1 |
| LjT | BNL | 41.30 | 1.79 | 0.04 | 0 | 0 | 166.34 | 4.69 | 1 |
| LjT | BZM | 18.64 | 1.36 | 0.09 | 0 | 2 | 212.26 | 3.27 | 4 |
| LjT | C09704 | 29.56 | 1.37 | 0.06 | 1 | 1 | 222.41 | 4.56 | 7 |
| LjT | Caeruloside C | 55.64 | -2.91 | 0.73 | 7 | 15 | 550.57 | -3.70 | 11 |
| LjT | Caeruloside C_qt | 5.40 | -1.64 | 0.37 | 4 | 10 | 388.41 | -1.95 | 8 |
| LjT | Caffeate | 54.97 | 0.27 | 0.05 | 3 | 4 | 180.17 | 1.37 | 2 |
| LjT | CAM | 67.17 | 1.29 | 0.05 | 0 | 1 | 152.26 | 1.94 | 0 |
| LjT | Caprylic acid | 16.40 | 0.90 | 0.02 | 1 | 2 | 144.24 | 2.72 | 6 |
| LjT | Cedrol | 16.23 | 1.35 | 0.12 | 1 | 1 | 222.41 | 3.16 | 0 |
| LjT | Centauroside | 4.37 | -2.95 | 0.43 | 8 | 19 | 758.80 | -3.11 | 16 |
| LjT | Centauroside_qt | 55.79 | -0.84 | 0.50 | 2 | 9 | 434.48 | 0.38 | 10 |
| LjT | Chlorogenic acid | 11.93 | -1.03 | 0.33 | 6 | 9 | 354.34 | -0.42 | 5 |
| LjT | Chrysoeriol | 35.85 | 0.39 | 0.27 | 3 | 6 | 300.28 | 2.32 | 2 |
| LjT | CIS-2-PENTENOL | 66.40 | 1.02 | 0.00 | 1 | 1 | 86.15 | 1.12 | 2 |
| LjT | Copaene | 29.47 | 1.81 | 0.12 | 0 | 0 | 204.39 | 4.17 | 1 |
| LjT | Corymbosin | 51.96 | 0.88 | 0.41 | 1 | 7 | 358.37 | 2.80 | 5 |
| LjT | Cosmetin | 9.68 | -1.08 | 0.74 | 6 | 10 | 432.41 | 0.43 | 4 |
| LjT | D-Camphene | 34.98 | 1.81 | 0.04 | 0 | 0 | 136.26 | 2.93 | 0 |
| LjT | Dehydroxymorroniside | 20.69 | -1.25 | 0.46 | 4 | 10 | 388.41 | -2.25 | 5 |
| LjT | Dehydroxymorroniside_qt | 29.40 | 0.12 | 0.10 | 1 | 5 | 226.25 | -0.51 | 2 |
| LjT | Desaspidinol-A | 30.51 | 0.64 | 0.05 | 2 | 4 | 182.19 | 1.02 | 2 |
| LjT | Dinethylsecologanoside | 48.46 | -1.37 | 0.48 | 4 | 12 | 434.44 | -0.79 | 10 |
| LjT | Dinethylsecologanoside_qt | 4.50 | -0.21 | 0.12 | 1 | 7 | 272.28 | 0.39 | 7 |
| LjT | Disacoside B | 1.99 | -3.95 | 0.03 | 13 | 22 | 1075.39 | -0.41 | 12 |
| LjT | Disacoside B_qt | 15.83 | 0.17 | 0.74 | 3 | 4 | 472.78 | 5.33 | 2 |
| LjT | Dodekan | 17.74 | 1.79 | 0.02 | 0 | 0 | 170.38 | 5.85 | 9 |
| LjT | Eriodictyol-7-o-glucoside | 17.57 | -1.34 | 0.78 | 7 | 11 | 450.43 | 0.13 | 4 |
| LjT | Eriodyctiol (flavanone) | 41.35 | 0.05 | 0.24 | 4 | 6 | 288.27 | 2.03 | 1 |
| LjT | ETHYL FURAN | 55.07 | 1.68 | 0.01 | 0 | 1 | 96.14 | 1.75 | 1 |
| LjT | Ethyl linolenate | 46.10 | 1.54 | 0.20 | 0 | 2 | 306.54 | 6.55 | 15 |
| LjT | Ethylpalmitate | 18.99 | 1.41 | 0.14 | 0 | 2 | 284.54 | 6.97 | 16 |
| LjT | Eudesmol | 35.38 | 1.28 | 0.09 | 1 | 1 | 224.43 | 4.10 | 1 |
| LjT | Eugenol | 56.24 | 1.35 | 0.04 | 1 | 2 | 164.22 | 2.55 | 3 |
| LjT | Farnesene | 17.42 | 1.95 | 0.05 | 0 | 0 | 204.39 | 5.52 | 7 |
| LjT | Farnesol | 28.44 | 1.32 | 0.06 | 1 | 1 | 222.41 | 4.76 | 7 |
| LjT | Farnesol acetate | 21.97 | 1.44 | 0.11 | 0 | 2 | 264.45 | 5.14 | 9 |
| LjT | Flavone der. | 27.12 | 0.83 | 0.27 | 1 | 5 | 298.31 | 2.84 | 3 |
| LjT | Fulvotomentoside A | 3.30 | -4.60 | 0.02 | 15 | 26 | 1207.52 | -1.65 | 14 |
| LjT | Geraniol | 23.93 | 1.19 | 0.02 | 1 | 1 | 154.28 | 2.93 | 4 |
| LjT | Germacrene D | 19.22 | 1.83 | 0.06 | 0 | 0 | 204.39 | 5.14 | 1 |
| LjT | Ginnol | 11.33 | 1.46 | 0.43 | 1 | 1 | 424.89 | 12.37 | 26 |
| LjT | GLO | 24.44 | -1.93 | 0.03 | 5 | 6 | 180.18 | -2.68 | 5 |
| LjT | Guaiol | 38.77 | 1.36 | 0.09 | 1 | 1 | 222.41 | 3.91 | 1 |
| LjT | Hederagenol | 22.42 | 0.10 | 0.74 | 3 | 4 | 472.78 | 5.33 | 2 |
| LjT | Helixin | 9.63 | -1.81 | 0.14 | 7 | 12 | 751.07 | 3.24 | 6 |
| LjT | HEPTACOSANE | 8.18 | 1.88 | 0.36 | 0 | 0 | 380.83 | 12.69 | 24 |
| LjT | Heptadecyloxirane | 12.44 | 1.67 | 0.15 | 0 | 1 | 282.57 | 7.61 | 16 |
| LjT | Heptenal | 37.16 | 1.30 | 0.01 | 0 | 1 | 112.19 | 2.29 | 4 |
| LjT | Hexadienal | 38.55 | 1.25 | 0.01 | 0 | 1 | 96.14 | 1.39 | 2 |
| LjT | Hexanal | 55.71 | 1.25 | 0.01 | 0 | 1 | 100.18 | 1.85 | 4 |
| LjT | Hexenal | 46.01 | 1.29 | 0.01 | 0 | 1 | 98.16 | 1.83 | 3 |
| LjT | Hexene | 60.01 | 1.78 | 0.00 | 0 | 0 | 84.18 | 2.72 | 3 |
| LjT | Hyacinthin | 38.65 | 1.31 | 0.02 | 0 | 1 | 120.16 | 1.52 | 2 |
| LjT | Hydnocarpin | 2.06 | -0.13 | 0.94 | 4 | 9 | 464.45 | 3.21 | 4 |
| LjT | Hydroquinone | 29.26 | 0.89 | 0.02 | 2 | 2 | 110.12 | 1.30 | 0 |
| LjT | HYKOP | 32.79 | 0.25 | 0.05 | 3 | 4 | 182.19 | 1.39 | 3 |
| LjT | Hyperin | 6.94 | -1.42 | 0.77 | 8 | 12 | 464.41 | -0.59 | 4 |
| LjT | Indole | 34.38 | 1.81 | 0.03 | 1 | 0 | 117.16 | 2.12 | 0 |
| LjT | Inositol | 18.86 | -1.52 | 0.05 | 6 | 6 | 180.18 | -3.06 | 0 |
| LjT | Loniceracetalides B | 5.48 | -1.44 | 0.63 | 4 | 12 | 476.53 | -0.17 | 9 |
| LjT | Loniceracetalides B_qt | 61.19 | -0.09 | 0.19 | 1 | 7 | 314.37 | 1.00 | 6 |
| LjT | Isobutyl tiglate | 24.51 | 1.30 | 0.02 | 0 | 2 | 156.25 | 2.76 | 4 |
| LjT | Isochlorogenic acid C | 1.78 | -1.32 | 0.69 | 7 | 12 | 516.49 | 1.56 | 9 |
| LjT | Isochlorogenic,acid | 1.79 | -1.10 | 0.69 | 7 | 12 | 516.49 | 1.56 | 9 |
| LjT | Isothiazole, trimethyl- | 67.44 | 1.42 | 0.02 | 0 | 1 | 127.23 | 1.76 | 0 |
| LjT | Junipene | 44.07 | 1.82 | 0.11 | 0 | 0 | 204.39 | 4.18 | 0 |
| LjT | Kaempferol | 41.88 | 0.26 | 0.24 | 4 | 6 | 286.25 | 1.77 | 1 |
| LjT | Kryptoxanthin | 47.25 | 1.69 | 0.57 | 1 | 1 | 552.96 | 10.76 | 10 |
| LjT | Lauric acid | 23.59 | 1.02 | 0.04 | 1 | 2 | 200.36 | 4.54 | 10 |
| LjT | L-Bornyl acetate | 65.52 | 1.29 | 0.08 | 0 | 2 | 196.32 | 2.35 | 2 |
| LjT | Leukol | 35.25 | 1.59 | 0.03 | 0 | 1 | 129.17 | 2.02 | 0 |
| LjT | Lignoceric acid | 14.90 | 1.24 | 0.33 | 1 | 2 | 368.72 | 10.02 | 22 |
| LjT | Linalool | 49.37 | 0.86 | 0.04 | 1 | 2 | 170.28 | 1.43 | 4 |
| LjT | LINALOOL (D) | 38.29 | 1.29 | 0.02 | 1 | 1 | 154.28 | 2.74 | 4 |
| LjT | Loganic | 34.96 | -0.58 | 0.05 | 4 | 5 | 176.19 | -0.59 | 1 |
| LjT | Loganic acid | 4.92 | -1.81 | 0.40 | 6 | 10 | 376.40 | -2.33 | 4 |
| LjT | Loganic acid_qt | 114.65 | -0.59 | 0.09 | 3 | 5 | 214.24 | -0.58 | 1 |
| LjT | Loganin | 5.90 | -1.48 | 0.44 | 5 | 10 | 390.43 | -2.08 | 5 |
| LjT | Loniceracetalide A | 28.29 | -1.31 | 0.58 | 4 | 11 | 460.53 | -1.16 | 8 |
| LjT | Loniceracetalide A_qt | 89.38 | -0.30 | 0.17 | 1 | 6 | 298.37 | 0.59 | 5 |
| LjT | Loniceracetalide B | 10.77 | -1.37 | 0.58 | 4 | 11 | 460.53 | -1.16 | 8 |
| LjT | Loniceracetalide B_qt | 89.28 | 0.16 | 0.17 | 1 | 6 | 298.37 | 0.59 | 5 |
| LjT | Luteolin | 36.16 | 0.19 | 0.25 | 4 | 6 | 286.25 | 2.07 | 1 |
| LjT | Luteolin-7-o-glucoside | 7.29 | -1.23 | 0.78 | 7 | 11 | 448.41 | 0.16 | 4 |
| LjT | Macranthoidin A | 4.15 | -4.97 | 0.01 | 16 | 27 | 1237.55 | -2.16 | 15 |
| LjT | Macranthoidin B | 6.69 | -5.80 | 0.01 | 19 | 32 | 1399.71 | -3.91 | 18 |
| LjT | Madreselvin A | 6.03 | -2.50 | 0.59 | 10 | 17 | 640.60 | -2.08 | 8 |
| LjT | Madreselvin B | 3.01 | -2.42 | 0.26 | 12 | 20 | 788.72 | -0.36 | 11 |
| LjT | Mandenol | 42.00 | 1.46 | 0.19 | 0 | 2 | 308.56 | 6.99 | 16 |
| LjT | Methional | 53.62 | 1.04 | 0.00 | 0 | 1 | 104.19 | 0.55 | 3 |
| LjT | methyl (1R,4aS,6S,7R,7aS)-1,6-dihydroxy-7-methyl-1,4a,5,6,7,7a-hexahydrocyclopenta[d]pyran-4-carboxylate | 29.99 | -0.36 | 0.10 | 2 | 5 | 228.27 | -0.33 | 2 |
| LjT | Methyl caffeate | 30.68 | 0.54 | 0.06 | 2 | 4 | 194.20 | 1.62 | 3 |
| LjT | Methyl chlorogenate | 9.97 | -0.74 | 0.36 | 5 | 9 | 368.37 | -0.16 | 6 |
| LjT | Methyl isomyristate | 20.26 | 1.38 | 0.08 | 0 | 2 | 242.45 | 5.50 | 12 |
| LjT | METHYL LINOLEATE | 41.93 | 1.44 | 0.17 | 0 | 2 | 294.53 | 6.64 | 15 |
| LjT | Methyl myristate | 19.68 | 1.36 | 0.08 | 0 | 2 | 242.45 | 5.71 | 13 |
| LjT | Methyl octadeca-8,11-dienoate | 41.93 | 1.46 | 0.17 | 0 | 2 | 294.53 | 6.64 | 15 |
| LjT | Methyl palmitate | 18.09 | 1.37 | 0.12 | 0 | 2 | 270.51 | 6.62 | 15 |
| LjT | Methyl-9-methyl tetradecanoate | 13.70 | 1.35 | 0.09 | 0 | 2 | 256.48 | 5.96 | 13 |
| LjT | Methyllinolenate | 46.15 | 1.48 | 0.17 | 0 | 2 | 292.51 | 6.20 | 14 |
| LjT | Methyl-p-coumarate | 20.14 | 0.83 | 0.05 | 1 | 3 | 178.20 | 1.89 | 3 |
| LjT | Muurolene | 19.50 | 1.84 | 0.08 | 0 | 0 | 204.39 | 4.75 | 1 |
| LjT | Myristic acid | 21.18 | 1.07 | 0.07 | 1 | 2 | 228.42 | 5.46 | 12 |
| LjT | Neochlorogenic acid | 10.65 | -1.43 | 0.33 | 5 | 9 | 353.33 | -1.09 | 5 |
| LjT | Nerol | 35.66 | 1.15 | 0.02 | 1 | 1 | 154.28 | 2.93 | 4 |
| LjT | Neryl acetate | 57.47 | 1.25 | 0.04 | 0 | 2 | 196.32 | 3.31 | 6 |
| LjT | NON | 26.74 | 0.96 | 0.03 | 1 | 2 | 172.30 | 3.63 | 8 |
| LjT | Nonacosanol | 10.57 | 1.48 | 0.43 | 1 | 1 | 424.89 | 12.38 | 27 |
| LjT | Nonanal | 40.28 | 1.31 | 0.02 | 0 | 1 | 142.27 | 3.22 | 7 |
| LjT | Nonanoic acid | 40.51 | 0.92 | 0.02 | 1 | 2 | 158.27 | 3.17 | 7 |
| LjT | Ochnaflavone | 2.54 | -0.11 | 0.57 | 5 | 10 | 538.48 | 4.67 | 4 |
| LjT | Octadecylglycol | 16.18 | 1.07 | 0.19 | 1 | 2 | 314.62 | 7.23 | 19 |
| LjT | Octanol | 21.06 | 1.16 | 0.01 | 1 | 1 | 130.26 | 2.80 | 6 |
| LjT | Olean-12-en-28-oic acid, 23-hydroxy-3-((O-beta-D-xylopyranosyl-(1-3)-O-6-deoxy-alpha-L-mannopyranosyl-(1-2)-alpha-L-arabinopyranosyl)oxy)-, (3beta,4alpha)- | 3.74 | -2.34 | 0.06 | 9 | 16 | 883.20 | 2.00 | 8 |
| LjT | Oleanolic acid | 29.02 | 0.59 | 0.76 | 2 | 3 | 456.78 | 6.42 | 1 |
| LjT | o-Thymol | 43.28 | 1.58 | 0.03 | 1 | 1 | 150.24 | 3.24 | 1 |
| LjT | p-Coumaric acid | 43.29 | 0.46 | 0.04 | 2 | 3 | 164.17 | 1.64 | 2 |
| LjT | PEL | 44.03 | 1.11 | 0.02 | 1 | 1 | 122.18 | 1.55 | 2 |
| LjT | Pent-3-en-2-one | 50.20 | 1.25 | 0.00 | 0 | 1 | 84.13 | 0.86 | 1 |
| LjT | Pentadecene | 17.72 | 1.84 | 0.05 | 0 | 0 | 210.45 | 6.82 | 12 |
| LjT | PHB | 30.15 | 0.39 | 0.03 | 2 | 3 | 138.13 | 1.17 | 1 |
| LjT | PHYTANTRIOL | 23.20 | 0.45 | 0.18 | 3 | 3 | 330.62 | 5.47 | 14 |
| LjT | Phytofluene | 43.18 | 2.29 | 0.50 | 0 | 0 | 543.02 | 14.10 | 19 |
| LjT | Prenal | 48.87 | 1.23 | 0.00 | 0 | 1 | 84.13 | 1.36 | 1 |
| LjT | Propyl vinyl ketone | 72.27 | 1.35 | 0.01 | 0 | 1 | 98.16 | 1.58 | 3 |
| LjT | Protocatechuic acid | 25.37 | 0.10 | 0.04 | 3 | 4 | 154.13 | 0.90 | 1 |
| LjT | PTL | 59.53 | 1.21 | 0.00 | 0 | 1 | 86.15 | 1.40 | 3 |
| LjT | Quercetin-3-o-β-D-glu | 1.81 | -1.40 | 0.79 | 8 | 13 | 478.39 | -0.38 | 4 |
| LjT | Quinic acid | 55.92 | -1.79 | 0.06 | 4 | 6 | 191.18 | -3.07 | 1 |
| LjT | Rhoifolin | 6.68 | -1.87 | 0.77 | 8 | 14 | 578.57 | -0.43 | 6 |
| LjT | SCG | 23.59 | -1.58 | 0.36 | 4 | 10 | 388.41 | -1.98 | 8 |
| LjT | Scolymoside | 3.84 | -2.16 | 0.73 | 9 | 15 | 594.57 | -0.70 | 6 |
| LjT | Secologanate | 17.56 | -1.90 | 0.33 | 5 | 10 | 374.38 | -2.23 | 7 |
| LjT | Secologanic acid_qt | 73.14 | -0.51 | 0.07 | 2 | 5 | 212.22 | -0.49 | 4 |
| LjT | Secologanic dibutylacetal | 20.05 | -0.97 | 0.67 | 4 | 11 | 546.73 | 1.84 | 18 |
| LjT | Secologanic dibutylacetal_qt | 53.65 | 0.34 | 0.29 | 1 | 6 | 384.57 | 3.58 | 15 |
| LjT | Secologanin dimethylacetal_qt | 2.71 | -0.07 | 0.11 | 1 | 6 | 272.33 | 0.01 | 7 |
| LjT | Secologanin_qt | 33.33 | -0.10 | 0.08 | 1 | 5 | 226.25 | -0.23 | 5 |
| LjT | Secologanoside | 26.92 | -2.31 | 0.37 | 4 | 11 | 388.36 | -3.64 | 7 |
| LjT | Secologanoside 7-methylester | 3.88 | -1.66 | 0.45 | 5 | 12 | 420.41 | -1.04 | 9 |
| LjT | Secologanoside 7-methylester_qt | 38.01 | -0.37 | 0.11 | 2 | 7 | 258.25 | 0.13 | 6 |
| LjT | Secologanoside_qt | 79.21 | -1.53 | 0.09 | 1 | 6 | 226.20 | -1.89 | 4 |
| LjT | Secoxyloganin | 3.79 | -1.63 | 0.39 | 5 | 11 | 404.41 | -2.03 | 5 |
| LjT | Secoxyloganin_qt | 34.80 | -0.32 | 0.09 | 2 | 6 | 242.25 | -0.28 | 1 |
| LjT | Sitogluside | 20.63 | -0.14 | 0.62 | 4 | 6 | 576.95 | 6.34 | 9 |
| LjT | Stigmasterol | 43.83 | 1.44 | 0.76 | 1 | 1 | 412.77 | 7.64 | 5 |
| LjT | Stigmasterol-β-glucoside | 2.40 | -0.23 | 0.63 | 4 | 6 | 574.93 | 5.89 | 8 |
| LjT | Succinic acid | 29.62 | -0.44 | 0.01 | 2 | 4 | 118.10 | -0.41 | 3 |
| LjT | Sulcatone | 26.36 | 1.35 | 0.01 | 0 | 1 | 126.22 | 1.79 | 3 |
| LjT | Sweroside aglycone | 68.68 | 0.15 | 0.08 | 1 | 4 | 196.22 | 0.15 | 1 |
| LjT | Tetradecane | 15.94 | 1.79 | 0.04 | 0 | 0 | 198.44 | 6.76 | 11 |
| LjT | Thymol | 41.47 | 1.60 | 0.03 | 1 | 1 | 150.24 | 3.24 | 1 |
| LjT | Tricin | 27.86 | 0.51 | 0.34 | 3 | 7 | 330.31 | 2.30 | 3 |
| LjT | Ursolic acid | 16.77 | 0.67 | 0.75 | 2 | 3 | 456.78 | 6.47 | 1 |
| LjT | Vogeloside | 5.62 | -1.17 | 0.46 | 4 | 10 | 388.41 | -1.78 | 5 |
| LjT | WLN: NCR B1 | 55.48 | 1.61 | 0.02 | 0 | 1 | 117.16 | 2.19 | 0 |
| LjT | WLN: QR BQ DQ | 22.93 | 0.59 | 0.02 | 3 | 3 | 126.12 | 1.03 | 0 |
| LjT | WLN: VHR | 32.63 | 1.32 | 0.01 | 0 | 1 | 106.13 | 1.59 | 1 |
| LjT | XYLOSTOSIDINE | 43.17 | -1.07 | 0.64 | 4 | 9 | 415.51 | -1.66 | 4 |
| LjT | XYLOSTOSIDINE_qt | 4.01 | 0.34 | 0.15 | 1 | 4 | 253.35 | 0.09 | 1 |
| LjT | Zeaxanthin | 21.17 | 1.22 | 0.54 | 2 | 2 | 568.96 | 9.53 | 10 |
| LjT | ZINC03978781 | 43.83 | 1.32 | 0.76 | 1 | 1 | 412.77 | 7.64 | 5 |
| LjT | Zingiberene | 18.80 | 1.91 | 0.06 | 0 | 0 | 204.39 | 5.08 | 4 |
| LjT | α cadinene | 18.73 | 1.85 | 0.08 | 0 | 0 | 204.39 | 4.75 | 1 |
| LjT | α-Cubebene | 16.73 | 1.83 | 0.11 | 0 | 0 | 204.39 | 4.17 | 1 |
| LjT | β-Carotene | 37.18 | 2.25 | 0.58 | 0 | 0 | 536.96 | 12.00 | 1 |
| LjT | β-Citronellol | 38.89 | 1.20 | 0.02 | 1 | 1 | 156.30 | 3.05 | 5 |
| LjT | β-Cubebene | 32.81 | 1.83 | 0.11 | 0 | 0 | 204.39 | 4.22 | 1 |
| LjT | β-Rhodinol | 38.05 | 1.19 | 0.02 | 1 | 1 | 156.30 | 3.05 | 5 |
| LjT | γ-Muurolene | 21.53 | 1.84 | 0.08 | 0 | 0 | 204.39 | 4.80 | 1 |
| LjT | δ-Amorphene | 17.95 | 1.85 | 0.08 | 0 | 0 | 204.39 | 4.94 | 1 |
| LjT/AcT | Furol | 34.35 | 1.08 | 0.01 | 0 | 2 | 96.09 | 0.99 | 1 |
| LjT/AcT | Heriguard | 11.93 | -1.03 | 0.33 | 6 | 9 | 354.34 | -0.42 | 5 |
| LjT/AcT | Quercetin | 46.43 | 0.05 | 0.28 | 5 | 7 | 302.25 | 1.50 | 1 |
| LjT/AcT | Rutin | 3.20 | -1.93 | 0.68 | 10 | 16 | 610.57 | -1.45 | 6 |
| LjT/AcT | β-caryophyllene | 29.70 | 1.83 | 0.09 | 0 | 0 | 204.39 | 4.75 | 0 |
| LjT/AcT | β-Elemene | 25.63 | 1.84 | 0.06 | 0 | 0 | 204.39 | 4.79 | 3 |
| LjT/AcT/Cm | β-Sitosterol | 36.91 | 1.32 | 0.75 | 1 | 1 | 414.79 | 8.08 | 6 |
| LjT/Cm | Palmitic acid | 19.30 | 1.09 | 0.10 | 1 | 2 | 256.48 | 6.37 | 14 |
| AcT | (-)-nopinene | 44.84 | 1.80 | 0.05 | 0 | 0 | 136.26 | 2.93 | 0 |
| AcT | (2R,3R)-3,5-dihydroxy-2-(4-hydroxyphenyl)-7-methoxychroman-4-one | 24.84 | 0.14 | 0.26 | 3 | 6 | 302.30 | 2.00 | 2 |
| AcT | (E)-3-[4-hydroxy-3-[(E)-4-hydroxy-3-methyl-but-2-enyl]-5-(3-methylbut-2-enyl)phenyl]acrylic acid | 23.76 | 0.41 | 0.19 | 3 | 4 | 316.43 | 4.26 | 7 |
| AcT | (E)-3-[4-hydroxy-3-[(Z)-4-hydroxy-3-methyl-but-2-enyl]-5-(3-methylbut-2-enyl)phenyl]acrylic acid | 12.86 | 0.35 | 0.19 | 3 | 4 | 316.43 | 4.26 | 7 |
| AcT | (L)-alpha-Terpineol | 48.80 | 1.39 | 0.03 | 1 | 1 | 154.28 | 2.42 | 1 |
| AcT | 2-NONANONE | 8.51 | 1.34 | 0.02 | 0 | 1 | 142.27 | 2.70 | 6 |
| AcT | 4'-Methylcapillarisin | 72.18 | 0.57 | 0.35 | 2 | 7 | 330.31 | 3.06 | 6 |
| AcT | 5-Hydroxyferulate | 59.99 | 0.24 | 0.07 | 3 | 5 | 210.20 | 1.35 | 3 |
| AcT | 7-Methylcapillarisin | 5.08 | 0.51 | 0.34 | 2 | 7 | 330.31 | 3.06 | 4 |
| AcT | Arcapillin | 48.96 | 0.60 | 0.41 | 3 | 8 | 360.34 | 2.29 | 4 |
| AcT | Artepillin A | 68.32 | 0.45 | 0.24 | 2 | 4 | 316.43 | 3.82 | 6 |
| AcT | Artepillin C | 38.39 | 0.83 | 0.17 | 2 | 3 | 300.43 | 5.35 | 6 |
| AcT | Ayapanin | 41.55 | 0.97 | 0.06 | 0 | 3 | 176.18 | 1.88 | 1 |
| AcT | Azelex | 16.90 | -0.04 | 0.04 | 2 | 4 | 188.25 | 1.87 | 8 |
| AcT | Butal | 68.66 | 1.18 | 0.00 | 0 | 1 | 72.12 | 0.94 | 2 |
| AcT | Capillanol | 62.02 | 1.29 | 0.04 | 1 | 1 | 174.26 | 2.97 | 2 |
| AcT | Capillarin | 87.01 | 1.36 | 0.08 | 0 | 2 | 198.23 | 3.00 | 1 |
| AcT | Capillarisin | 57.56 | 0.49 | 0.31 | 3 | 7 | 316.28 | 2.81 | 3 |
| AcT | Capillarol | 37.41 | 0.54 | 0.12 | 1 | 4 | 260.31 | 3.17 | 5 |
| AcT | Capillene | 47.19 | 2.11 | 0.03 | 0 | 0 | 154.22 | 3.95 | 1 |
| AcT | Capillin | 52.44 | 1.50 | 0.04 | 0 | 1 | 168.20 | 3.38 | 1 |
| AcT | Car-3-ene | 45.15 | 1.85 | 0.04 | 0 | 0 | 136.26 | 2.87 | 0 |
| AcT | Cirsilineol | 4.81 | 0.77 | 0.37 | 2 | 7 | 344.34 | 2.55 | 4 |
| AcT | Cirsimaritin | 30.35 | 0.72 | 0.30 | 2 | 6 | 314.31 | 2.57 | 3 |
| AcT | Demethoxycapillarisin | 52.33 | 0.31 | 0.25 | 3 | 6 | 286.25 | 2.83 | 2 |
| AcT | D-limonene | 38.80 | 1.82 | 0.02 | 0 | 0 | 136.26 | 3.50 | 1 |
| AcT | Eugenol | 56.24 | 1.35 | 0.04 | 1 | 2 | 164.22 | 2.55 | 3 |
| AcT | Eupalitin | 46.11 | 0.62 | 0.33 | 3 | 7 | 330.31 | 2.01 | 3 |
| AcT | Eupatolitin | 42.55 | 0.16 | 0.37 | 4 | 8 | 346.31 | 1.74 | 3 |
| AcT | Genkwanin | 37.13 | 0.63 | 0.24 | 2 | 5 | 284.28 | 2.59 | 2 |
| AcT | Hirsutrin | 1.86 | -1.66 | 0.77 | 8 | 12 | 464.41 | -0.59 | 4 |
| AcT | Isoarcapillin | 57.40 | 0.40 | 0.41 | 3 | 8 | 360.34 | 2.29 | 4 |
| AcT | Isorhamnetin | 49.60 | 0.31 | 0.31 | 4 | 7 | 316.28 | 1.76 | 2 |
| AcT | Isorhamnetin-3-mono-β-D-glucoside | 4.11 | -1.35 | 0.80 | 7 | 12 | 478.44 | -0.34 | 5 |
| AcT | Isorhamnetin-3-O-glucoside | 1.17 | -1.24 | 0.80 | 7 | 12 | 478.44 | -0.34 | 5 |
| AcT | Isoscopoletin | 23.46 | 0.71 | 0.08 | 1 | 4 | 192.18 | 1.62 | 1 |
| AcT | Methyleugenol | 73.36 | 1.47 | 0.04 | 0 | 2 | 178.25 | 2.81 | 4 |
| AcT | Neocapillene | 24.45 | 2.09 | 0.03 | 0 | 0 | 154.22 | 3.95 | 0 |
| AcT | Norcapillene | 38.06 | 2.03 | 0.03 | 0 | 0 | 140.19 | 4.00 | 1 |
| AcT | OXL | 29.68 | -0.64 | 0.01 | 2 | 4 | 90.04 | -0.48 | 1 |
| AcT | Penta-1,3-diynylbenzene | 15.35 | 2.07 | 0.03 | 0 | 0 | 140.19 | 3.50 | 0 |
| AcT | Piceol | 36.80 | 0.87 | 0.03 | 1 | 2 | 136.16 | 1.30 | 1 |
| AcT | Rhamnocitrin | 12.90 | 0.48 | 0.27 | 3 | 6 | 300.28 | 2.02 | 2 |
| AcT | Salicylic acid | 32.13 | 0.63 | 0.03 | 2 | 3 | 138.13 | 1.17 | 1 |
| AcT | Scoparone | 74.75 | 0.85 | 0.09 | 0 | 4 | 206.21 | 1.87 | 2 |
| AcT | Scopoletin | 27.32 | 0.73 | 0.08 | 1 | 4 | 192.18 | 1.62 | 1 |
| AcT | Scopoletol | 27.77 | 0.71 | 0.08 | 1 | 4 | 192.18 | 1.62 | 1 |
| AcT | Vanillin | 52.00 | 0.68 | 0.03 | 1 | 3 | 152.16 | 1.31 | 2 |
| AcT | α-Humulene | 39.81 | 1.87 | 0.06 | 0 | 0 | 204.39 | 5.04 | 0 |
| Cm | (2R,3R,4S)-2-(6-aminopurin-9-yl)-4-(hydroxymethyl)oxolan-3-ol | 38.44 | -0.90 | 0.16 | 4 | 7 | 251.28 | -1.48 | 2 |
| Cm | (2R,3S,5S)-5-(6-aminopurin-9-yl)-2-(hydroxymethyl)oxolan-3-ol | 30.13 | -1.10 | 0.15 | 4 | 7 | 251.28 | -1.25 | 2 |
| Cm | 20-Hexadecanoylingenol | 28.20 | 0.30 | 0.68 | 3 | 6 | 586.94 | 7.38 | 17 |
| Cm | Adenine | 62.81 | -0.30 | 0.03 | 3 | 4 | 135.15 | -0.58 | 0 |
| Cm | Arachidonic acid | 45.57 | 1.20 | 0.20 | 1 | 2 | 304.52 | 6.41 | 14 |
| Cm | Caffeine | 89.46 | 0.58 | 0.08 | 0 | 5 | 194.22 | -0.10 | 0 |
| Cm | Cerevisterol | 39.52 | 0.35 | 0.77 | 3 | 3 | 432.76 | 5.26 | 4 |
| Cm | Cholesteryl palmitate | 31.05 | 1.45 | 0.45 | 0 | 2 | 625.19 | 14.35 | 21 |
| Cm | Cinnamaldehyde | 31.99 | 1.35 | 0.02 | 0 | 1 | 132.17 | 1.95 | 2 |
| Cm | CLR | 37.87 | 1.43 | 0.68 | 1 | 1 | 386.73 | 7.38 | 5 |
| Cm | Cordycedipeptide A | 63.12 | -0.34 | - | 4 | 6 | 227.30 | -0.65 | 4 |
| Cm | Cordycepin | 36.83 | -1.13 | - | 4 | 7 | 251.28 | -1.45 | 2 |
| Cm | Cordylagenin | 17.36 | 0.34 | 0.78 | 2 | 4 | 432.71 | 3.66 | 0 |
| Cm | D-Mannoheptulose | 29.74 | -2.02 | 0.05 | 6 | 7 | 210.21 | -3.20 | 6 |
| Cm | EIC | 41.90 | 1.16 | 0.14 | 1 | 2 | 280.50 | 6.39 | 14 |
| Cm | Ergosterol | 14.29 | 1.47 | 0.72 | 1 | 1 | 396.72 | 6.93 | 4 |
| Cm | Galactomannan | 10.92 | -3.92 | 0.70 | 11 | 16 | 504.50 | -6.01 | 7 |
| Cm | GLB | 47.71 | -1.89 | 0.04 | 5 | 6 | 180.18 | -2.51 | 1 |
| Cm | GUP | 43.04 | -1.82 | 0.04 | 5 | 6 | 180.18 | -2.51 | 1 |
| Cm | Isoergotamine | 8.10 | 0.32 | 0.21 | 3 | 9 | 581.73 | 3.12 | 4 |
| Cm | LFA | 8.46 | 1.83 | 0.13 | 0 | 0 | 282.62 | 9.50 | 17 |
| Cm | Linoleic | 41.90 | 1.23 | 0.14 | 1 | 2 | 280.50 | 6.39 | 14 |
| Cm | Linoleyl acetate | 42.10 | 1.36 | 0.20 | 0 | 2 | 308.56 | 6.85 | 16 |
| Cm | MTL | 17.73 | -1.58 | 0.03 | 6 | 6 | 182.20 | -2.94 | 5 |
| Cm | NCA | 71.13 | 0.44 | 0.02 | 2 | 3 | 122.14 | -0.32 | 1 |
| Cm | Nicotinic acid | 47.65 | 0.34 | 0.02 | 1 | 3 | 123.12 | 0.28 | 1 |
| Cm | Oleic acid | 33.13 | 1.14 | 0.14 | 1 | 2 | 282.52 | 6.84 | 15 |
| Cm | Peroxyergosterol | 44.39 | 0.86 | 0.82 | 1 | 3 | 428.72 | 6.73 | 4 |
| Cm | Stearic acid | 17.83 | 1.15 | 0.14 | 1 | 2 | 284.54 | 7.28 | 16 |
| Cm | Styrone | 38.35 | 1.14 | 0.02 | 1 | 1 | 134.19 | 1.69 | 2 |
| Cm | TGL | 15.13 | 0.54 | 0.13 | 0 | 6 | 891.67 | 22.26 | 56 |
| Cm | Thiamine | 19.87 | -0.32 | 0.11 | 3 | 4 | 265.40 | -0.05 | 4 |
| Cm | TRE | 2.32 | -3.08 | 0.24 | 8 | 11 | 342.34 | -4.26 | 4 |
| Cm | Uracil | 42.53 | 0.05 | 0.02 | 2 | 4 | 112.10 | -1.01 | 0 |
| Cm | Uralene | 11.70 | 0.63 | 0.49 | 4 | 7 | 384.41 | 3.43 | 4 |
| Cm | Uridine | 10.49 | -1.14 | 0.11 | 4 | 8 | 244.23 | -2.45 | 2 |
| Cm | Vitamin C | 13.34 | -0.86 | 0.04 | 4 | 6 | 176.14 | -1.76 | 2 |
| Cm | Vitamin G | 6.79 | -1.22 | 0.50 | 5 | 10 | 376.41 | 0.23 | 5 |

LjT, *Lonicera japonica* Thunberg; AcT, *Artemisia capillaris* Thunberg; Cm, *Cordyceps militaris*; MW, molecular weight;
OB, oral bioavailability; Caco-2, Caco-2 cell permeability; DL, drug-likeness score; Hdon, number of hydrogen bond donors;
Hacc, number of hydrogen bond acceptors; MW, molecular weight; AlogP, octanol-water partition coefficient log P;
RBN, number of rotatable bonds. **Supplementary Table S2. List of active phytochemical components of FDY003.**

| Herbal medicines | Phytochemical components | OB | Caco-2 | DL | Hdon | Hacc | MW | AlogP | RBN |
| --- | --- | --- | --- | --- | --- | --- | --- | --- | --- |
| LjT | Chrysoeriol | 35.85 | 0.39 | 0.27 | 3 | 6 | 300.28 | 2.32 | 2 |
| LjT | Corymbosin | 51.96 | 0.88 | 0.41 | 1 | 7 | 358.37 | 2.80 | 5 |
| LjT | Eriodyctiol (flavanone) | 41.35 | 0.05 | 0.24 | 4 | 6 | 288.27 | 2.03 | 1 |
| LjT | Loniceracetalides B_qt | 61.19 | -0.09 | 0.19 | 1 | 7 | 314.37 | 1.00 | 6 |
| LjT | Kaempferol | 41.88 | 0.26 | 0.24 | 4 | 6 | 286.25 | 1.77 | 1 |
| LjT | Luteolin | 36.16 | 0.19 | 0.25 | 4 | 6 | 286.25 | 2.07 | 1 |
| LjT/AcT | Quercetin | 46.43 | 0.05 | 0.28 | 5 | 7 | 302.25 | 1.50 | 1 |
| LjT/AcT/Cm | β-Sitosterol | 36.91 | 1.32 | 0.75 | 1 | 1 | 414.79 | 8.08 | 6 |
| AcT | 4'-Methylcapillarisin | 72.18 | 0.57 | 0.35 | 2 | 7 | 330.31 | 3.06 | 6 |
| AcT | Arcapillin | 48.96 | 0.60 | 0.41 | 3 | 8 | 360.34 | 2.29 | 4 |
| AcT | Artepillin A | 68.32 | 0.45 | 0.24 | 2 | 4 | 316.43 | 3.82 | 6 |
| AcT | Capillarisin | 57.56 | 0.49 | 0.31 | 3 | 7 | 316.28 | 2.81 | 3 |
| AcT | Cirsimaritin | 30.35 | 0.72 | 0.30 | 2 | 6 | 314.31 | 2.57 | 3 |
| AcT | Demethoxycapillarisin | 52.33 | 0.31 | 0.25 | 3 | 6 | 286.25 | 2.83 | 2 |
| AcT | Eupalitin | 46.11 | 0.62 | 0.33 | 3 | 7 | 330.31 | 2.01 | 3 |
| AcT | Eupatolitin | 42.55 | 0.16 | 0.37 | 4 | 8 | 346.31 | 1.74 | 3 |
| AcT | Genkwanin | 37.13 | 0.63 | 0.24 | 2 | 5 | 284.28 | 2.59 | 2 |
| AcT | Isoarcapillin | 57.40 | 0.40 | 0.41 | 3 | 8 | 360.34 | 2.29 | 4 |
| AcT | Isorhamnetin | 49.60 | 0.31 | 0.31 | 4 | 7 | 316.28 | 1.76 | 2 |
| Cm | Cordycepin | 36.83 | -1.13 | - | 4 | 7 | 251.28 | -1.45 | 2 |

LjT, *Lonicera japonica* Thunberg; AcT, *Artemisia capillaris* Thunberg; Cm, *Cordyceps militaris*; MW, molecular weight;
OB, oral bioavailability; Caco-2, Caco-2 cell permeability; DL, drug-likeness score; Hdon, number of hydrogen bond donors;
Hacc, number of hydrogen bond acceptors; MW, molecular weight; AlogP, octanol-water partition coefficient log P;
RBN, number of rotatable bonds.

**Supplementary Table S3. List of targets of active phytochemical components of FDY003.**

| Herbal medicines | Phytochemical components | Targets |
| --- | --- | --- |
| LjT | Chrysoeriol | ABCC1*, CREB1, CYP1B1*, PTPRS, XDH |
| LjT | Corymbosin | ABCG2*, CREB1, CYP1B1*, PTPRS |
| LjT | Eriodyctiol (flavanone) | ABCB1, ABCC1*, ADIPOQ*, APOB*, BDNF, CA12*, CA4*, CA7, CBR1, CCK, CCL2*, CD69, CD86*, CYP19A1*, CYP1A2*, CYP1B1*, CYP3A4*, HMGA1, HMGCS2, HMOX1*, HSD17B1, KCNH2, KCNMA1, LDLR, PGF, PPARA, RAPGEF1, SCD, SCD5, SHBG, TAS2R31, TLR2* |
| LjT | Kaempferol | ABCB1, ABCC1*, ABCG2*, AHR*, AKR1B1, AKT1*, ALOX12, ALOX5*, AR*, ATM*, BCHE, CA12*, CA2*, CA7, CASP3*, CASP9*, CCL2*, CDK1*, CDK2*, CHUK*, CISD1, CSF2*, CTDSP1, CYP1A1*, CYP1A2*, CYP1B1*, CYP2B6, CYP2D6, CYP3A4*, DAPK1*, DIO2, EGFR*, ESR1, ESR2*, ESRRA, F2*, FLT3, GSTP1*, H2AFX, HCK, HMOX1*, HSD17B1, HSD17B2, IGF1R*, IGF2*, IL2*, ITGA2, JUN*, MAPK1*, MAPK3*, MMP1*, MMP2*, MPO*, NFKBIA*, NOS1, NOS2*, NOX4, NR1I2, NR1I3, P4HB, PIM1, PTGES, PTPRS, RB1*, RPS6KA3, SLC2A1, SRC*, STAT1*, STAT3*, TNFRSF11B, TP53*, TYR, UGT1A1, UGT1A10, UGT1A3, UGT1A7*, UGT1A8, UGT1A9, UGT3A1, XDH |
| LjT | Luteolin | ABCC1*, ABCG2*, ADAMTS3, ADAMTS4, ADORA1, AGT, AKR1B1, AKR1B10, AKT1*, ALOX15, ALOX5*, APP*, ARG1, AURKB*, BCL2L1*, C3, CA12*, CA2*, CA4*, CA7, CASP3*, CASP7, CASP9*, CCNA2, CCNB1*, CCNB2, CCNB3, CD38, CDH1*, CDK1*, CDK2*, CDK4*, CDK5R1, CDK6, CREB1, CSNK2A1, CSNK2A2, CSNK2B, CYP19A1*, CYP1A1*, CYP1A2*, CYP1B1*, E2F5, EGFR*, ERBB2*, ESR2*, EZH2, FLT3, FN1, FOS*, FOSB, FOXO1, GLO1, GPR35, GSK3B*, HMOX1*, HSP90AA1*, IGF1*, IGF2*, IL1B*, IRS1*, JUN*, JUNB, JUND, LCN2, MAOA, MAP3K8, MAPK1*, MAPK10, MAPK3*, MAPK8, MAPK9, MMP12, MMP2*, MMP9*, MTOR*, NFE2L2*, NOS1, NOS2*, NOX4, PARP1, PCK1, PKM, PPARG*, PTK2*, PTPRS, RPS6KA1, RPS6KA2, RPS6KA3, SMAD2, STAT3*, SYK, TAF9, TBK1, TLR4, TLR5, TNFRSF10B*, TNKS, TNKS2, TOP1, TP53*, TTR, UGT1A3, USP8, VEGFA*, VRK1, XDH |
| LjT/AcT | Quercetin | ABCA1, ABCB1, ABCC1*, ABCC4*, ABCC5, ABCG2*, ACACA, ADIPOQ*, ADORA1, ADORA2A, AHR*, AIFM1, AKR1A1, AKR1B1, AKR1C1, AKR1C2, AKR1C3, AKR1C4, AKT1*, ALK*, ALOX12, ALOX15, ALOX5*, AOX1, APAF1, APOB*, AR*, ARNT, ATP2A1, ATP5A1, ATP5B, ATP5C1, AURKB*, AVPR2, AXL*, BACE1*, BAX*, BCL2*, BDNF, BID, CA1*, CA12*, CA13, CA14, CA2*, CA3, CA4*, CA5A, CA6, CA7, CA9, CAMK2B, CASP3*, CASP7, CASP8, CASP9*, CAT*, CCL2*, CCR4, CD38, CD97, CDK1*, CDK2*, CDKN1A*, CDKN2A*, CFTR*, CHEK2*, CHUK*, CKB, CSF2*, CSNK2A1, CTNNB1*, CTRL, CXCL10, CXCR1, CXCR4*, CYBB, CYCS, CYP19A1*, CYP1A1*, CYP1A2*, CYP1B1*, CYP2C8, CYP2C9, CYP2D6, CYP2E1, CYP3A4*, DAPK1*, DIABLO, DIO2, DRD4, EDN1, EEF1A1, EGFR*, EIF2A, EIF2AK2, ELAVL1, ERN1, ERN2, ESR1, ESR2*, F2*, F3*, FAU, FLT3, FNDC5, FOS*, FOXM1, GADD45A*, GLO1, GLRA1, GPR35, GSK3B*, GSTP1*, HCK, HIBCH, HIF1A*, HIST3H3, HMOX1*, HPGDS, HSD17B2, HSPA1A*, HSPA4, HSPB1*, ICAM1*, IGF1R*, IGFBP3*, IL15, IL17A, IL1B*, IL6*, IL8, JUN*, KCNMA1, KDR*, KRAS*, LEPR*, MAOA, MAPK1*, MAPK14, MAPK3*, MAPK8, MCL1, MET*, MMP1*, MMP13, MMP2*, MMP3*, MMP9*, MPO*, NAMPT, NEK2, NEK6, NFE2L2*, NFKBIA, NKX3-1, NOS1, NOS2*, NOS3*, NOX3, NOX4, NPC1L1, NPY1R, NR1I2, NR1I3, NT5E, NUAK1, ODC1, P4HB, PARP1*, PDX1*, PIK3CA*, PIK3CG, PIK3R1*, PIM1, PKN1*, PLA2G1B*, PLAT, PLAU*, PLK1*, PON1*, PON2, POR, PPARA, PRDX5, PTGS1, PTGS2*, PTK2*, PTPRS, PYGB, PYGL, PYGM, RAF1*, RB1*, RNASEL, RPS6KA5, SERPIND1, SESN2, SIRT1, SLC12A2, SLC2A1, SLC2A2, SLC2A4, SP1, SRC*, STAT1*, STK17B, SULT1A1, SULT1E1, TBK1, TFAM, TGFA, TGM2*, TLR1, TLR10, TLR2*, TLR4, TLR6*, TMPRSS11D, TNF*, TNFRSF10B*, TNFSF10*, TP53*, TRPM7, TYR, UGT1A1, UGT1A10, UGT1A3, UGT1A5, UGT1A7*, UGT1A8, UGT1A9, UGT2A3, UGT2B15, UGT2B4, UGT3A1, VEGFA*, XBP1, XDH |
| LjT/AcT/Cm | β-Sitosterol | ACBD7, CASP3*, CASP9*, CYP17A1*, DHPS, DRAP1, GPBAR1, ICAM1*, LFT, MDM2*, MEX3D, MTOR*, NPC1L1, NR1D2, PARP1*, RAC1, RANBP2, RUVBL1, TNNC1, USF1 |
| AcT | 4'-Methylcapillarisin | CREB1 |
| AcT | Arcapillin | CREB1, CYP1B1*, PTPRS |
| AcT | Artepillin A | CPSF4, CYP2R1*, EME1, FOLH1, PTPRJ, RNF31 |
| AcT | Capillarisin | CREB1, CYP1B1*, PTPRS |
| AcT | Cirsimaritin | ADORA1, ADORA2A, ADORA3, AKR1B1, CREB1, CYP1B1*, PTPRS |
| AcT | Eupalitin | CREB1, CYP1B1* |
| AcT | Eupatolitin | CREB1, CYP1B1* |
| AcT | Genkwanin | ALDH2*, CREB1, CYP1A1*, CYP1A2*, CYP1B1*, DUSP1, PTPRS, XDH |
| AcT | Isoarcapillin | CREB1, PTPRS |
| AcT | Isorhamnetin | ABCB1, ABCC1*, AKT1*, CA12*, CA2*, CA4*, CA7, CREB1, CYP1A1*, CYP1B1*, ERN1, HMOX1*, MAPK8, MAPK9, NOS2*, P4HB, PON2, PRKCD, XDH |
| Cm | Cordycepin | ADCY5, ADK, ADORA1, ADORA2A, ADORA2B, ADORA3, AHCY, AHCYL1, AHCYL2, AMD1, BCL7A, CASP3*, CASP8, CASP9*, DCK*, DGUOK, DNMT3B, DOT1L, DTYMK, FOXP3, GAPDH, HGF*, HSPA5, HSPA8*, IL10*, IL1B*, IL6*, IMPDH1, KMT5C, LEPR*, MMP9, MTAP, MYC*, NOS1, P2RX1, P2RY11, PAPOLA, PRMT7, QARS1, RARS1, RNASEL, SETDB1, SLC29A1*, SMS, SRM, STAR, TARS1, TK1*, TLR4, TMPRSS11D |

LjT, *Lonicera japonica* Thunberg; AcT, *Artemisia capillaris* Thunberg; Cm, *Cordyceps militaris*.

*, pancreatic cancer-associated targets.
